# In silico analysis of HLA-1 and HLA-2 recognition of a designed recombinant human papillomavirus vaccine based on L1 protein HPV subtype 45

**DOI:** 10.1186/s43141-023-00593-8

**Published:** 2023-12-13

**Authors:** Asri Sulfianti, Nihayatul Karimah, Astutiati Nurhasanah

**Affiliations:** Centre for Vaccine and Drug Research, National Research and Innovation Agency Republic of Indonesia, LAPTIAB 1, Gedung 611, Kawasan Puspiptek Serpong, Tangerang Selatan, Banten 15314 Indonesia

**Keywords:** HLA-1 alleles, HLA-2 alleles, Antigenic peptides, HPV, Recombinant vaccine, Immuno-informatics in silico analysis, T cells, Cervical cancer

## Abstract

**Background:**

Human leukocyte antigen (HLA) can bind and present the processed antigenic peptide derived from the vaccine to the T cell receptor, and this capability is crucial in determining the effectivity of the vaccine to terminate virus-infected cells, activate macrophages, and induce B cells to produce antibodies. A recombinant vaccine candidate based on protein L1 HPV45 was designed and analysed whether it is recognisable by T cells through the binding of their epitopes to HLAs.

**Methods:**

The study consisted of two parts: part one was the analysis of the L1 recombinant protein binding to HLA-1 and 2 epitopes, whereas part two was the distribution analysis of HPV-linked HLA allele. HLA allele sets found at high frequency in the general population and in specific Indonesian population were listed for the binding analysis of the recombinant L1 HPV45 protein. In part one, immunoepitope servers from IEDB were used to predict the binding of the designed proteins to HLA alleles. The prediction method for MHC-I binding prediction was the NetMHCpan EL 4.1 whilst for MHC-II binding prediction was the Consensus approach. Antigenicity analysis for each peptide was conducted using VaxiJen 2.0 with the threshold 1.0 to select the highly antigenic peptides, and positions of these epitopes in the secondary and tertiary structure of the recombinant protein were also predicted. The percent population coverage of the alleles capable of binding to these epitopes worldwide was also estimated. In part two, the worldwide distribution and frequency of HPV-related HLA-1 and 2 were studied.

**Result:**

Two highly antigenic peptides (EEYDLQFIF and KLKFWTVDLK) were recognised by high-frequency HLA-1 alleles in both, the general and Western Javanese. In addition to these two epitopes, a few more peptides are also recognised by the high-frequency Western Javanese HLA-1 alleles, which are not in Weiskopf’s list of high-frequency HLA-1 alleles in the general population. Analysis of the highly antigenic epitopes binding to HLA-DRB1 alleles in general (YIKGTSANM) and Western Javanese (LRRRPTIGP) populations showed that these peptide cores associate to HLA-DRB1*04, albeit the different sub-types, due to the presence of different allele in each population group. Analysis of the epitopes and the positive binding alleles showed on average 25.65% population coverage.

**Conclusion:**

The recombinant vaccine candidate based on protein L1 HPV45 is presumed to contain highly antigenic peptides that can bind to high-frequency HLA-1 and 2 alleles present in general and Western Javanese populations. It was expected that the protein is capable of eliciting T cell-mediated responses in both populations; however, in vitro study is needed to prove the protectiveness of the designed recombinant protein.

**Supplementary Information:**

The online version contains supplementary material available at 10.1186/s43141-023-00593-8.

## Background

Based on estimations for 2020, about 36,633 new cervical cancer cases are diagnosed annually in Indonesia [1]. With about 21,003 deaths occurring due to cervical cancer every year, the disease is the second leading cause of female cancer deaths in this country [1]

Cervical cancer is associated with human papillomavirus (HPV) infection, which causes cancer by knocking out tumour suppressor genes (p53 and pRb) via binding of E6 and E7 viral proteins to them. Several subtypes of this virus are categorised as ‘high’-risk genotypes. These subtypes include HPV 16, 18, 31, and 45 [2]. The HPV subtype 45 belongs to HPV18-related alpha-7 species and causes about 5% of all cervical cancer cases worldwide [3]. Somewhat similar prevalence was also observed in Indonesia. A study conducted in Surabaya, a city in Indonesia, in 2015, showed that the prevalence of HPV genotype in cervical adenocarcinoma and adenosquamous carcinoma was dominated by HPV types 18, 11, and 45, with frequencies 67.3, 10.9, and 5.5%, respectively [4].

Previously, we had designed a recombinant vaccine candidate based on the L1 protein of HPV45 [5]. L1 is a ~55 kD protein component of HPV capsid, with the ability to spontaneously self-assemble into virus-like particles (VLPs). The designed vaccine candidate differs only in two amino acids from the available L1 HPV45 sequence from Indonesia (GenBank: QRG45832.1) [6], and in three amino acids from the consensus L1 HPV sequence generated from the alignment of 63 L1 protein sequences submitted to NCBI from various countries between 1993 and 2019 [5]. Considering the close similarity between the designed recombinant vaccine and the online available sequences, it was expected that the vaccine will be able to elicit humoral responses similar to those produced by infection by HPV 45. However, long-term immunity towards HPV relies also on T cell-mediated responses, which in turn is influenced by the major histocompatibility complex (MHC) [7].

The MHC binds peptide fragments derived from pathogens and displays them on the cell surface for recognition by the appropriate T cells, causing termination of virus-infected cells, activation of macrophages to kill bacteria living in their intracellular vesicles, and activation of B cells to produce antibodies for elimination and neutralisation of extracellular pathogens [8]. Since the role of MHC, which in humans is also known as human leukocyte antigen (HLA), is to present processed antigenic peptides to the T cell receptor, its capability to bind and present peptides derived from the vaccine is crucial in determining the effectivity of the vaccine. In her review, Paaso et al. [9] classified several MHC molecules into two classes: those that were associated with persistent HPV infection and progress towards cervical cancer and those that were associated with clearance of the infection (Table 1).

**Table 1:** HLA-1 and 2 alleles associated with infection progress and clearance [9]

HLA-1		HLA-2	
Infection/progress	Clearance	Infection/progress	Clearance
HLA-A*02	HLA-B*14	HLA-DQA1*01:02	HLA-DQA1*01:03
HLA-A*02:01	HLA-B*15	HLA-DQA1*03	HLA-DQB1*02
HLA-A*03:01	HLA-B*15:01	HLA-DQA1*03:01	HLA-DQB1*05:01
HLA-A*31:01	HLA-B*27:05	HLA-DQB1*03	HLA-DQB1*06:03
HLA-A*33:03	HLA-B*35:03	HLA-DQB1*03:01	HLA-DQB1*06:04
HLA-B*07	HLA-B*40	HLA-DQB1*03:02	HLA-DRB1*09:01
HLA-B*35:01	HLA-B*40:06	HLA-DQB1*04:02	HLA-DRB1*13
HLA-B*37	HLA-B*52	HLA-DQB1*05	HLA-DRB1*13:01
HLA-B*37:01	HLA-C*01	HLA-DQB1*06:01	HLA-DRB1*13:02
HLA-B*39:01	HLA-C*03	HLA-DQB1*06:02	HLA-DRB1*13:10
HLA-B*44:02	HLA-C*08	HLA-DRB1*04	
HLA-B*58	HLA-Cw*02:02	HLA-DRB1*04:01	
HLA-B*58:01	HLA-Cw*04:01	HLA-DRB1*11	
HLA-C*07:02	HLA-G*01:03	HLA-DRB1*11:01	
HLA-Cw*05:01	HLA-G*01:04:01	HLA-DRB1*15	
HLA-Cw*07:04		HLA-DRB1*15:01	
HLA-G*01:01:02			
HLA-G*01:01:03			
HLA-G*01:01:05			
HLA-G*01:01:08			

Aiming to prove that the designed recombinant L1 HPV45 protein would be recognisable by T cells through binding between the epitopes with HLA-1 and HLA-2, T cell epitope exploration was performed. In this publication, we are presenting our analysis of the previously designed protein on HLA alleles. The analyses were performed in silico, based on computational analysis and literature search. Two sets of HLA alleles were used, one set containing the HLA alleles, covering 97 and 99% population for class I and class II, respectively [9–11], whereas the other set containing the HPV-related HLA alleles common in Indonesian population [9, 12]. This analysis is crucial because HLA polymorphisms had been shown before as an important risk determinant of HPV infection persistence and disease progression [9]. They play a central role in the immune recognition and subsequent clearance of virally infected cells. HLA-1 presents foreign antigens to CD8+ cytotoxic T lymphocytes, whereas HLA-2 presents antigens to the T cell receptor on the surface of Th cells [13]. Also, it is believed that the presence of HLA molecules that bind the HPV antigen with high affinity is associated with protection against cancer progression, whereas the presence of HLA molecules that do not recognise and bind HPV antigens is associated with increased risk of cervical pre-cancer stages and cancers [9].

## Methods

**Fig. 1:**
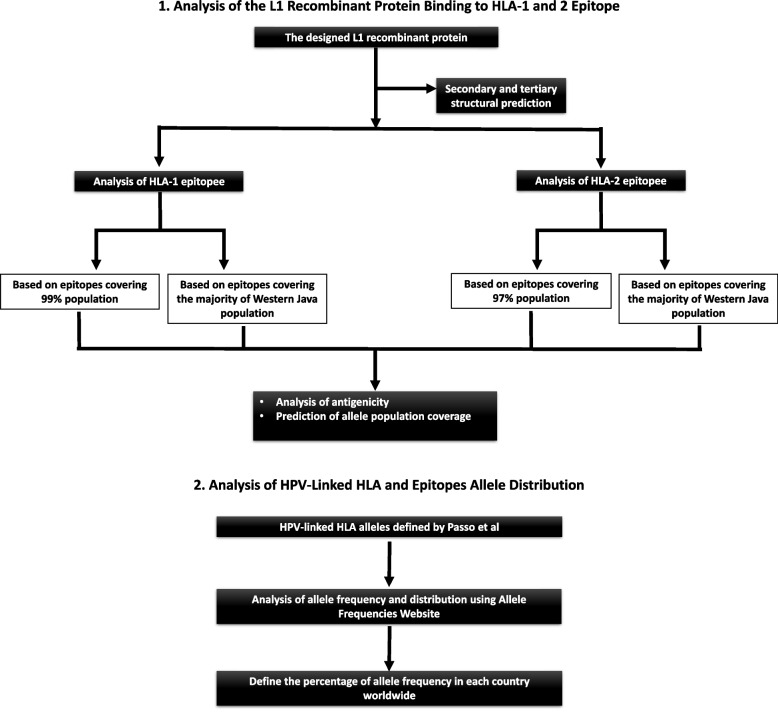
Schematic representation of the study workflow. Two parts were performed during the study. **A** Part one was the analysis of the L1 recombinant protein binding to HLA-1 and 2 Epitopes. **B** Part two was the distribution analysis of HPV-linked HLA allele

The study was performed in two parts: part one was the analysis of the L1 recombinant protein binding to HLA-1 and 2 epitopes, whereas part two was the distribution analysis of the HPV-linked HLA allele. The steps involved in the study are presented in Fig. 1.

### Recombinant protein L1HPV45

The recombinant protein L1HPV45 used in this study was designed previously by our group and had been published [5].

### Generation of the recombinant L1HPV45 protein 3D model

The secondary and tertiary structural prediction of the designed recombinant L1HPV45 protein was generated using the Robetta Prediction tool (https://robetta.bakerlab.org/) [14] (accessed on 3 March 2023), which performs a three-track network as the deep learning-based method called RoseTTAFold to integrate the protein folding. Robetta generated five models from the submitted sequence. The model with the least error estimation was chosen. The secondary structure was visualised using Protean-3D, whereas the position of the HLA epitopes in the modelled L1HPV45 structure was visualised using VMD [15].

### HLA allele sets

HLA-1 and HLA-2 allele sets found at high frequency in the general population were obtained from the Immune Epitope Database (IEDB) Analysis Resource (http://tools.iedb.org/mhci/ and http://tools.iedb.org/mhcii/), which were based on publications by Weiskopf et al. [11] and Greenbaum et al. [10], respectively. The HLA allele set found at high frequency in Indonesia was selected based on publication by Yuliwulandari et al. [12]. The HLA allele set related to HPV was selected based on publication by Paaso et al. [9].

### HLA allele inclusion

HPV-associated HLA-1 and HLA-2 alleles, as defined by Paaso et al. [9], which cover 97% of the world population [11] and 99% of the world population [10], respectively, were included in the binding analysis of the designed recombinant protein to determine the protein affinity to high-frequency alleles in the general population. HPV-associated alleles [9] present in the Indonesian population [12] were used in the binding analysis of the designed recombinant protein to determine the protein affinity to high-frequency alleles in the local (Indonesian) population.

### Prediction of HPV-related HLA allele distribution

Worldwide distribution of the HPV-related HLA alleles as classified by Paaso et al. [9] was predicted using the HLA-searching options in the Allele Frequency Net Database (http://www.allelefrequencies.net/hla.asp) [16] (sites accessed between 2 and 31 March 2023). The search was limited to gold and silver standard data only [17]. Allele frequencies were calculated with the same method as previously published by Zhou et al. [18]. The information on population data was obtained from sites of the World Bank Data (https://data.worldbank.org/indicator/SP.POP.TOTL), the World Population Reviews (https://worldpopulationreview.com/), and the United Nations’ 2022 Revision of World Population Prospects (https://population.un.org/wpp/ ).

### Immunoepitope predictions

Immunoepitope servers (http://tools.iedb.org/mhci/ and http://tools.iedb.org/mhcii/) were used to predict the binding of the designed proteins to HLA-1 and 2 alleles [19]. We used the IEDB recommended method for each HLA-1 and 2 allele predictions [20–22], where in HLA-1 allele prediction, peptide length was set at ‘all length’ (between 8 and 14 residues) [23], whereas, in HLA-2 allele prediction, it was set between 15 and 23 residues [22]. The IEDB Recommended tool in HLA-1 allele prediction was NetMHCpan EL 4.1 (site accessed on 26 January 2022). NetMHCpan is a method that using an artificial neural network generates quantitative predictions of the affinity of any peptide–MHC class I interaction [24]. The NetMHCpan EL 4.1 tool had been trained on data of mass spectrometry-eluted ligands [25]. The HLA-2 allele prediction used the Consensus approach [26, 27], which is the default recommended method from IEDB (site accessed on 2 February 2022). Using the Consensus approach, the top three methods that give the best predictions are selected for each MHC class II molecule, of which the binding can be predicted by three or more algorithms. For each method, the tested peptides are ranked based on their scores, where higher ranks indicate better binders. For each tested peptide, ranks from the three different methods are then taken and the median of the three is calculated. This median rank is taken as the consensus score [26]. The top three methods were selected from amongst the neural network-based alignment artificial algorithm (NN-align) [28], stabilisation matrix alignment method (SMM-align) [29], Combinatorial Library (CombLib) [30], and Sturniolo pocket profile [31], if any corresponding predictor was available for the molecule; otherwise, NetMHCIIpan [25] was used. The Consensus approach considers a combination of any three of the four methods, if available, where Sturniolo is used as a final choice.

### Antigenicity analysis

Antigenicity was analysed using VaxiJen 2.0 server (http://www.ddg-pharmfac.net/vaxijen/VaxiJen/VaxiJen.html), with antigenic threshold score set at 0.4 [32]. To select highly antigenic peptides, the threshold was raised to 1.0.

### Population coverage analysis

Analysis of the designed vaccine population coverage was performed with the population coverage analysis tool available on the IEDB website (http://tools.iedb.org/population/) (accessed between 2 and 31 March 2023) [33], using the same method as previously published by Bhattacharya [34].

## Results

### HLA allele inclusion

In this study, two sets of HLA alleles were included. Alleles from IEDB reference sets [10, 11], which had been classified by Paaso et al. [9] as HPV-related were included in the analysis of the recombinant protein binding to the high-frequency alleles in the general population. In the binding analysis of the recombinant protein to the high-frequency HLA alleles identified in Indonesia, we used HLA alleles from the population in West Java (as published by [12]), which had also been classified by Paaso et al. [9] as HPV-related HLA alleles. Since Java is the most populated island in Indonesia (56.11% population in 2020 [35]), the HLA alleles common in the island were expected to represent the actual allele diversity in the country’s population.

The alleles classified by Paaso as HPV-related alleles are presented in Table 1. The alleles used in the binding analysis of the protein to the West Java population are presented in (Table 2).

**Table 2:** HLA alleles used in the binding analysis of the recombinant protein to the high-frequency alleles identified in West Java [12]

HLA-1 Alleles		HLA-2 Alleles
HLA-A*02:01	HLA-B*07:02	DRB1*04:02
HLA-A*02:03	HLA-B*07:05	DRB1*04:03
HLA-A*02:06	HLA-B*15:01	DRB1*04:05
HLA-A*02:11	HLA-B*15:02	DRB1*04:06
HLA-A*03:01	HLA-B*15:10	DRB1*09:01
HLA-A*33:03	HLA-B*15:12	DRB1*11:01
	HLA-B*15:13	DRB1*13:02
	HLA-B*15:17	DRB1*15:01
	HLA-B*15:21	DRB1*15:02
	HLA-B*15:25	
	HLA-B*15:32	
	HLA-B*35:01	
	HLA-B*37:01	
	HLA-B*40:01	
	HLA-B*40:02	
	HLA-B*40:06	
	HLA-B*52:01	
	HLA-B*58:01	

### Global distribution of HPV-related HLA alleles

**Fig. 2:**
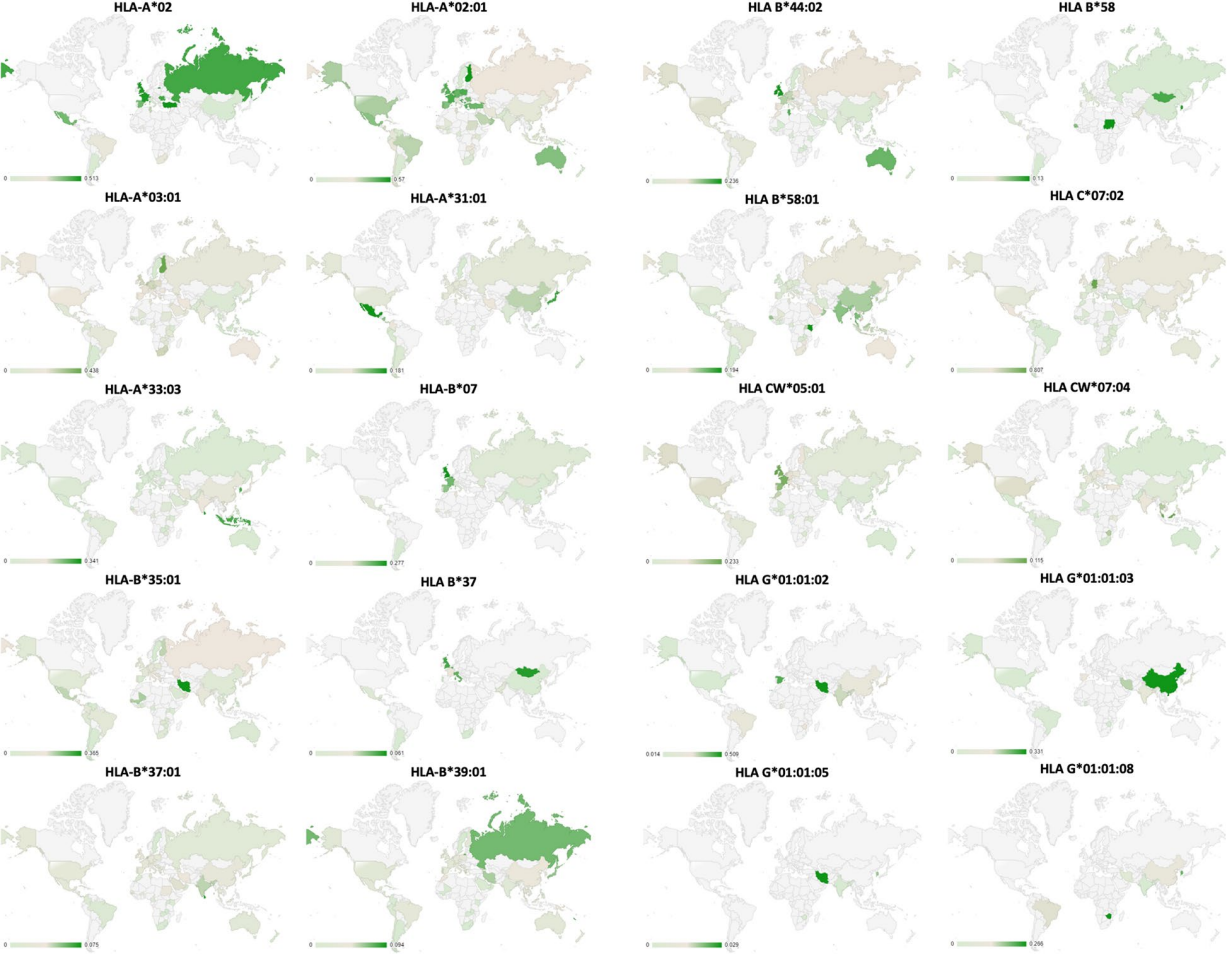
Distribution of HPV-related HLA-1 alleles (Paaso, 2019), which contribute to infection and progress of the disease. (Light grey indicates the area where no data was available)

**Fig. 3:**
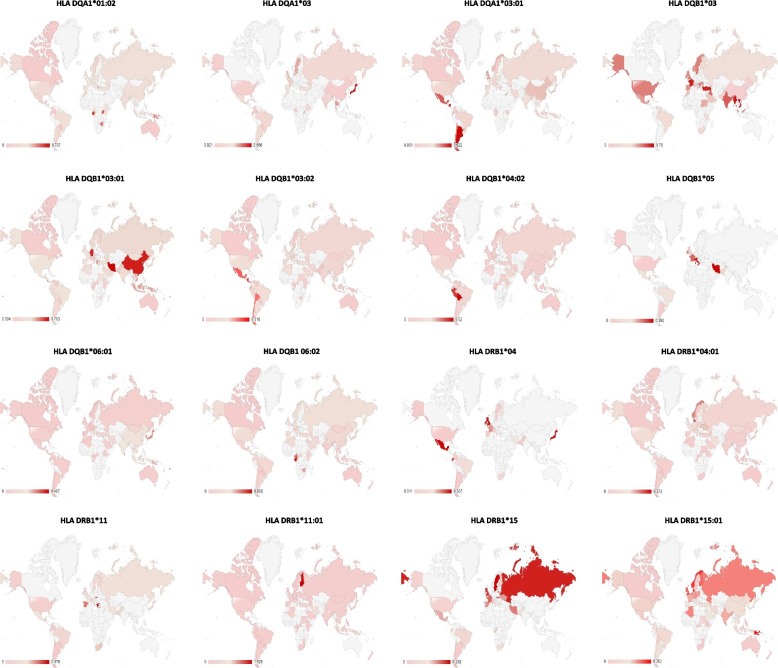
Distribution of HPV-related HLA-2 alleles, which contribute to infection and progress of the disease. (Light grey indicates the area where no data was available)

**Fig. 4:**
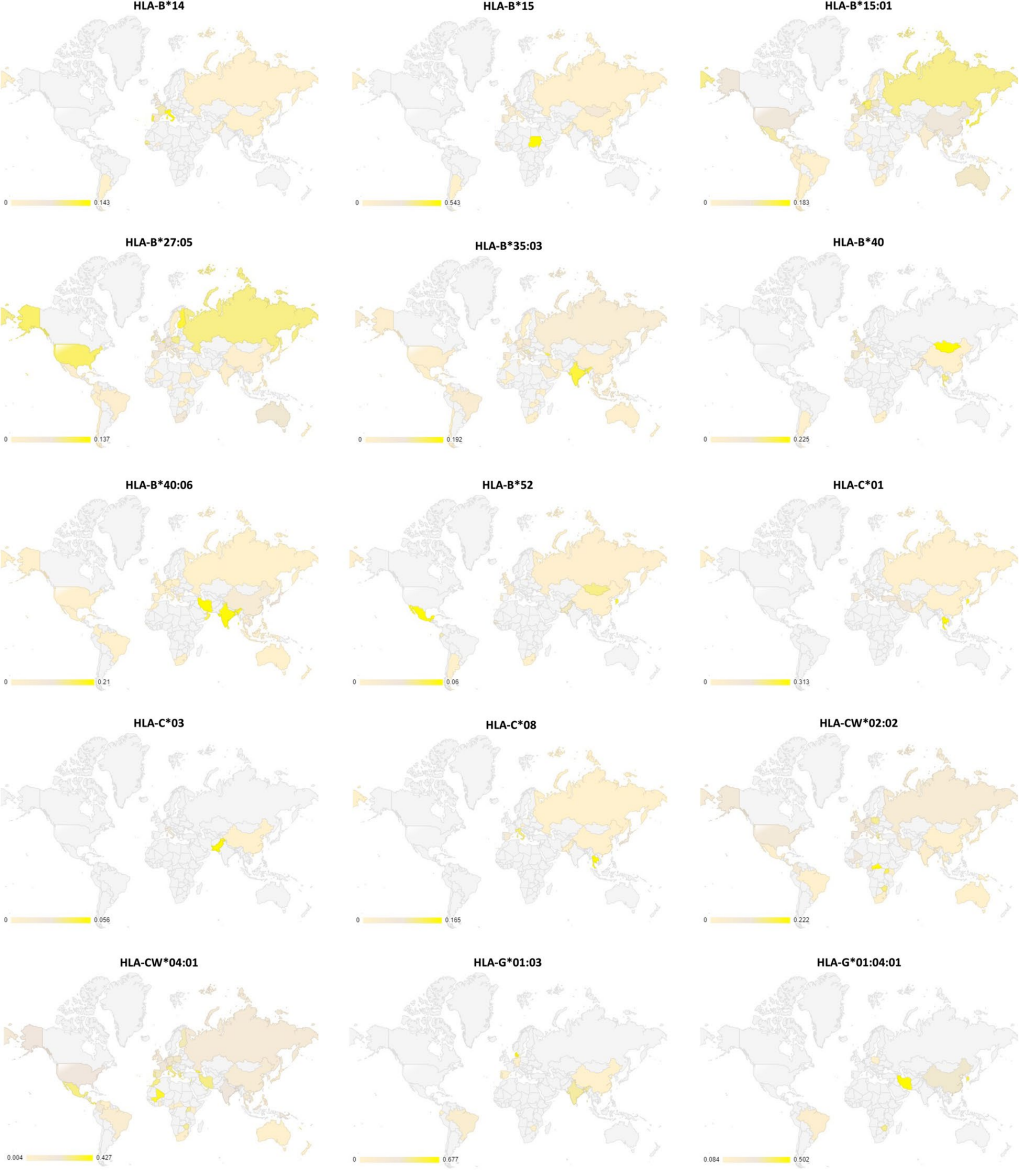
Distribution of HPV-related HLA-1 alleles, which contribute to clearance/regression of the disease. (Light grey indicates the area where no data was available)

**Fig. 5:**
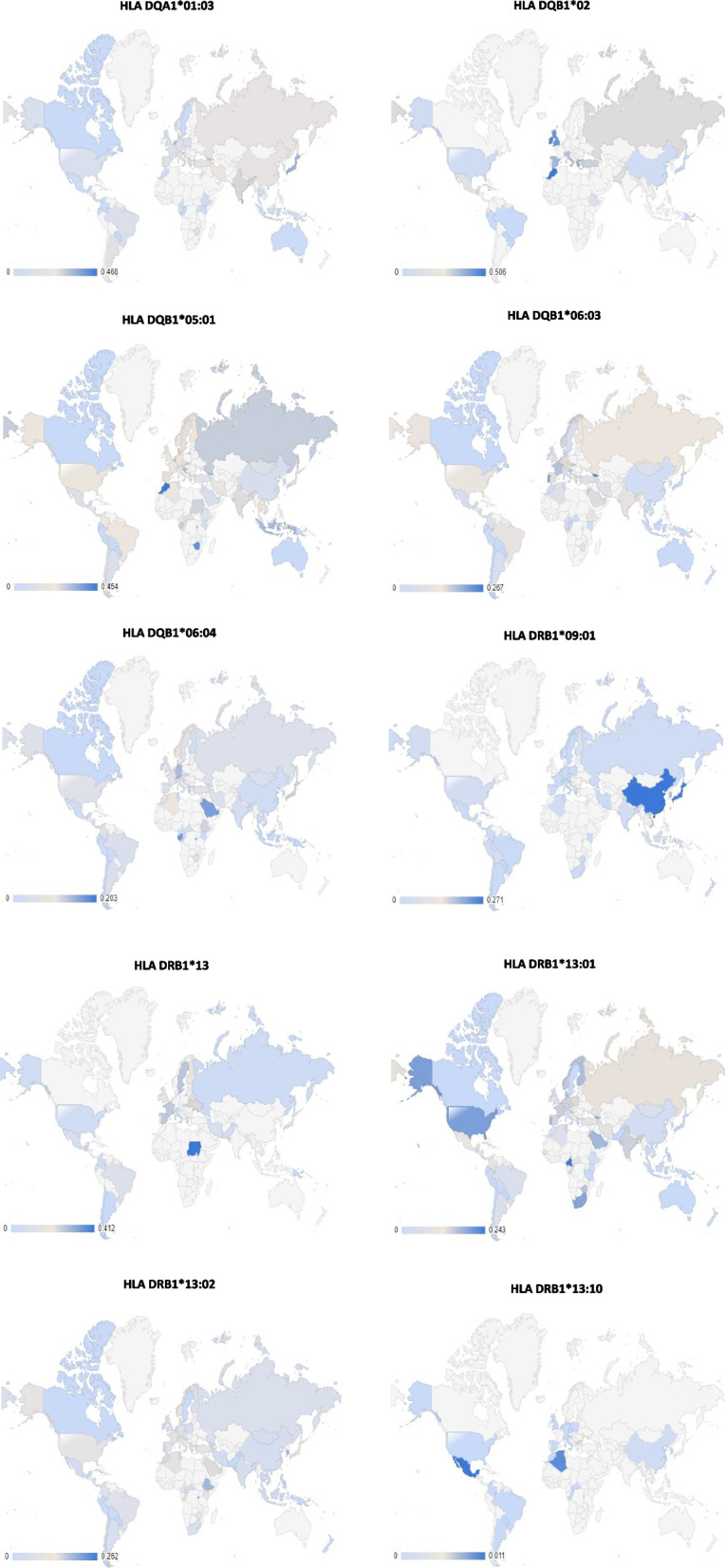
Distribution of HPV-related HLA-2 alleles, which contribute to the clearance/regression of the disease. (Light grey indicates the area where no data was available)

The distribution of the HPV-related HLA alleles globally is presented in Figs. 2–5, where Figs. 2 and 3 show the distribution of infection/progress-related HLA-1 and HLA-2 alleles, respectively, whilst Fig. 4 and 5 show the distribution of clearance-related HLA-1 and HLA-2 alleles, respectively. The allele frequencies in the graphs are colour-coded with low-intensity colour indicating lower frequency, which increases with increasing colour intensity.

### Binding of the recombinant protein to HPV-related high-frequency alleles in general population

Forty-nine peptides were selected, with a binding score ≥0.6, in the binding analysis of the recombinant protein with HLA-1 HPV-related high-frequency alleles in the general population. VaxiJen analysis was performed on these selected peptides (varying in length between 8 and 11 amino acids) to determine their antigenicity. The analysis showed that 20 peptides are antigenic, with VaxiJen scores between 0.41 and 1.74 (Supplementary Table 1), two of which are highly antigenic (VaxiJen scores > 1) (Table 3)*.*

**Table 3:** The binding sites of peptides to HPV-related high-frequency HLA-1 alleles in general population, with VaxiJen antigenicity scores >1

No	Peptide	Positions	Allele	VaxiJen Score
1	EEYDLQFIF	398–406	HLA-B*40:01	1.7384
2	KLKFWTVDLK	473–482	HLA-A*03:01	1.1434

Analysis of the recombinant protein in its binding to DRB1 selected 141 peptides (adjusted rank < 1), which were used for antigenicity analysis (Supplementary Table 2). The VaxiJen analysis indicated that 48 of them are antigenic, with VaxiJen scores lie between 0.4 and 1.104. The three most antigenic peptides (VaxiJen score >1) seem to have the same core and bind to the same allele (Table 4), with SMM IC50 slightly above 50.

**Table 4:** The binding sites of highly antigenic peptides, with VaxiJen antigenicity scores >1, and the HLA-DRB1 binding alleles from analysis of the HPV-related high-frequency HLA-DRB1 in the general population

No.	Peptide	Start-End	Length	HLA-2 Binding Allele	VaxiJen Score	IC50
1	DL**YIKGTSANM**RET	301–314	14	HLA-DRB1*04:01	1.104	55
2	DL**YIKGTSANM**RETP	301–315	15	HLA-DRB1*04:01	1.091	56
3	TDL**YIKGTSANM**RET	300–314	15	HLA-DRB1*04:01	1.0182	54

SMM core is shown in bold

### Binding of recombinant protein to HPV-related high-frequency HLA alleles in West Java

Ninety-five peptides, with a binding score ≥0.6, were selected in the binding analysis of the recombinant protein with HPV-related high-frequency HLA-1 alleles in West Java. These peptides, varying in length between 8 and 13 amino acids, were analysed further for their antigenicity using VaxiJen. The analysis indicated that 41 of these peptides were antigenic, with VaxiJen scores between 0.40 and 2.00 (Suplementary Table 3). Table 5 shows the selected highly antigenic peptides (VaxiJen score>1) and their HLA-1 binding sites. Two of these peptides bind more than one HLA-1 allele.

**Table 5:**
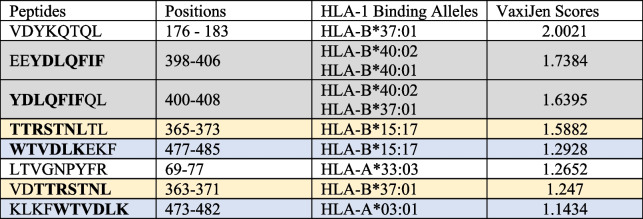
West Java originated HLA-1 binding sites of peptides with VaxiJen antigenicity scores >1

Peptides shaded with the same colours partially overlap to one another. Overlapping residues are in bold

In the binding analysis of the recombinant protein with HPV-related high-frequency DRB1 alleles in West Java, 162 peptides of 12–19 amino acids were selected, all with adjusted percentile rank <1. These selected peptides were then analysed further for their antigenicity using VaxiJen, which indicated that 68 of these peptides were antigenic (scores range 0.4–2.07) (Supplementary Table 4). Table 6 shows highly antigenic peptides (VaxiJen score >1) with their HLA-2 binding sites.

**Table 6:** West Java originated HLA-DRB1 binding sites of highly antigenic peptides

Peptides	Positions	HLA-2 binding alleles	VaxiJen scores
G**LRRRPTIGP**RKR	503–515	HLA-DRB1*04:02	2.0738
AG**LRRRPTIGP**RKR	502–515	HLA-DRB1*04:02	2.0411
**LRRRPTIGP**RKRP	504–516	HLA-DRB1*04:02	1.9788
VQAG**LRRRPTIGP**RKR	500–515	HLA-DRB1*04:02	1.8995
G**LRRRPTIGP**RKRP	503–516	HLA-DRB1*04:02	1.8611
AG**LRRRPTIGP**RKRP	502–516	HLA-DRB1*04:02	1.8494
VQAG**LRRRPTIGP**R	500–513	HLA-DRB1*04:02	1.8122
QAG**LRRRPTIGP**RKR	501–515	HLA-DRB1*04:02	1.8078
**LRRRPTIGP**RKRPA	504–517	HLA-DRB1*04:02	1.7785
AG**LRRRPTIGP**RK	502–514	HLA-DRB1*04:02	1.7631
AG**LRRRPTIGP**RKRPA	502–517	HLA-DRB1*04:02	1.6938
QAG**LRRRPTIGP**R	501–513	HLA-DRB1*04:02	1.6929
G**LRRRPTIGP**RKRPA	503–517	HLA-DRB1*04:02	1.6909
**LRRRPTIGP**RKRPAA	504–518	HLA-DRB1*04:02	1.6606
VQAG**LRRRPTIGP**RK	500–514	HLA-DRB1*04:02	1.6539
QAG**LRRRPTIGP**RKRP	501–516	HLA-DRB1*04:02	1.6522
G**LRRRPTIGP**RKRPAA	503–518	HLA-DRB1*04:02	1.5902
VQAG**LRRRPTIGP**	500–512	HLA-DRB1*04:02	1.5776
LVQAG**LRRRPTIGP**R	499–513	HLA-DRB1*04:02	1.5675
QAG**LRRRPTIGP**RK	501–514	HLA-DRB1*04:02	1.5305
**LRRRPTIGP**RKRPAAS	504–519	HLA-DRB1*04:02	1.5179
FLVQAG**LRRRPTIGP**R	498–513	HLA-DRB1*04:02	1.4645
LVQAG**LRRRPTIGP**RK	499–514	HLA-DRB1*04:02	1.441
LVQAG**LRRRPTIGP**	499–512	HLA-DRB1*04:02	1.3345
FLVQAG**LRRRPTIGP**	498–512	HLA-DRB1*04:02	1.244

The peptide core is shown in bold

### Generation of predicted secondary and tertiary structure

**Fig. 6:**
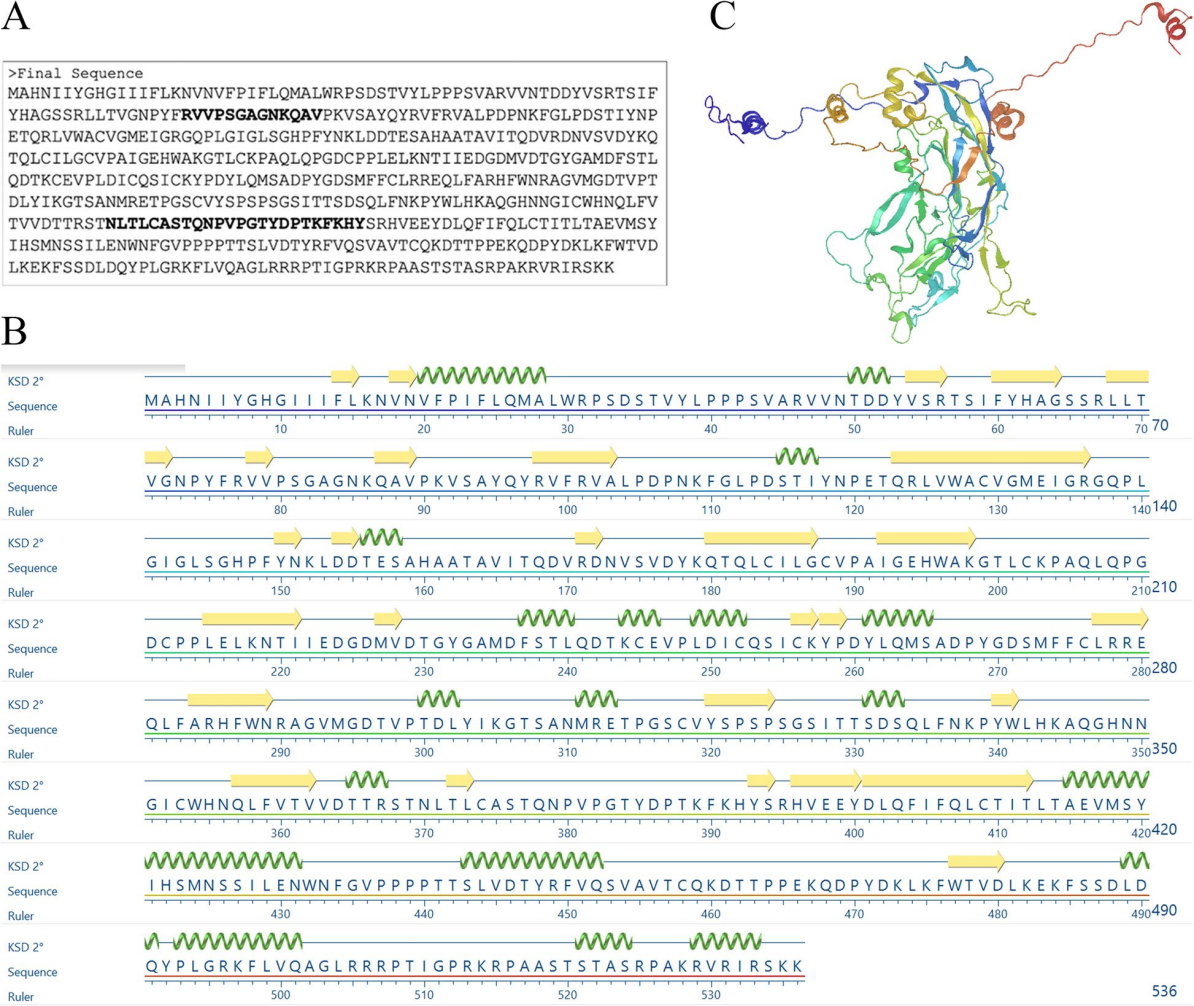
**A** Sequence of L1 HPV45 protein that had been previously optimised as a VLP vaccine candidate [5], **B** prediction of its secondary, and **C** tertiary structures

**Fig. 7:**
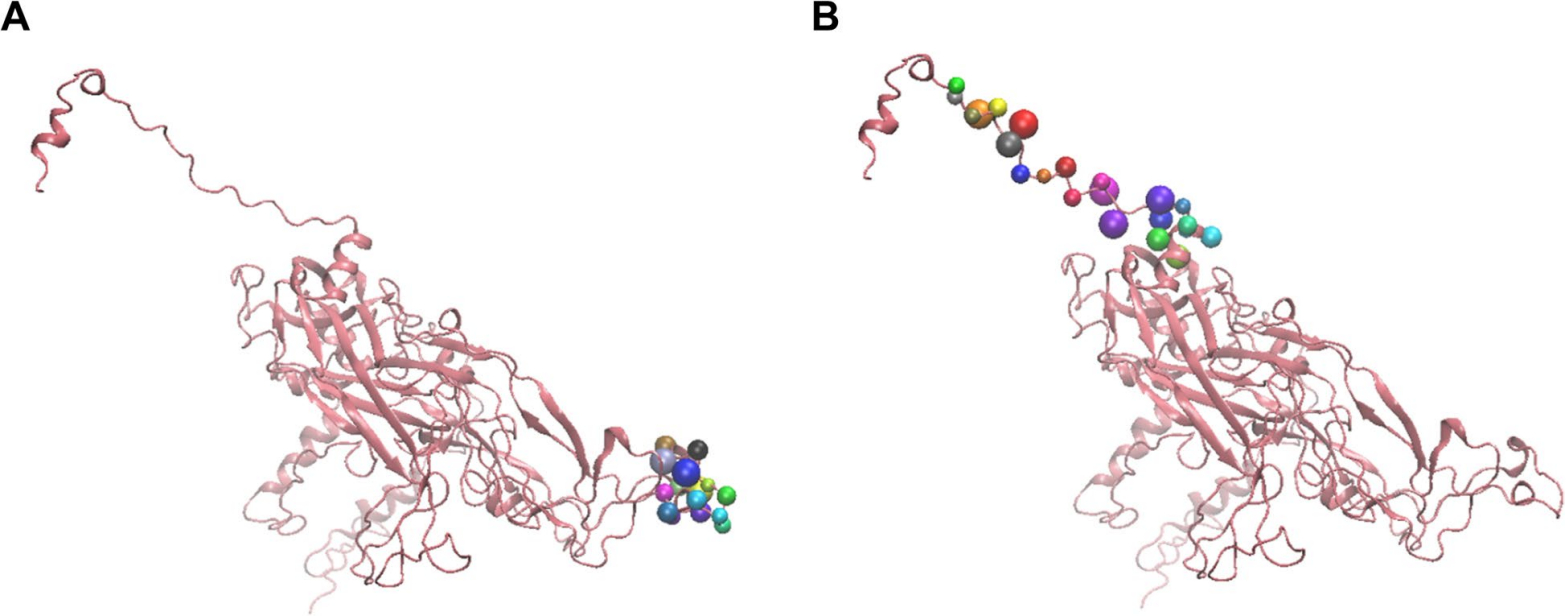
The position of antigenic peptides (shown in colorful beads) with VaxiJen antigenicity scores >1 which are predicted to bind with HLA-DRB1 from **A** general population (TDL**YIKGTSANM**RETP) and **B** West Java population (FLVQAG**LRRRPTIGP**RKRPAAS)

**Fig. 8:**
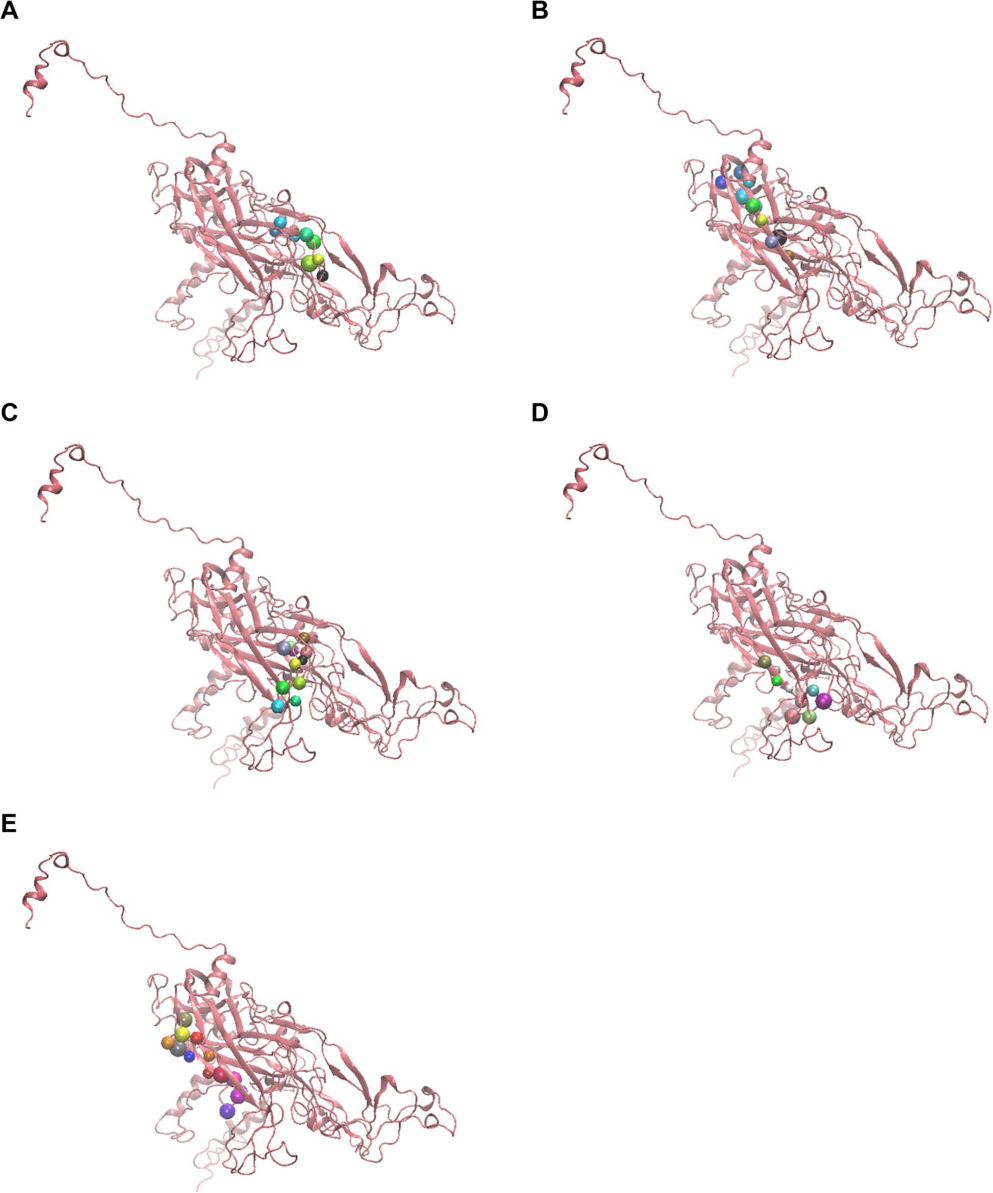
The position of antigenic peptides with VaxiJen antigenicity scores >1 which are predicted to bind with HLA-1 from the West Java population. The peptides shown in colorful beads are **A** VDYKQTQL, **B** EE**YDLQFIF**QL, **C** VD**TTRSTNL**TL, **D** LTVGNPYFR, and **E** KLKF**WTVDLK**EKF

The predicted secondary and tertiary structures of the protein are presented in Fig. 6 (B) and (C), respectively. Positions of the positive epitopes (VaxiJen antigencity scores >1), which are predicted to bind HLA-1, could be observed in Fig. 7, whereas Fig. 8 (A) (shown in colourful beads) shows the position of positive epitopes (VaxiJen antigencity scores >1), which are predicted to bind HLA-DRB1 from the general population (TDLYIKGTSANMRETP), and (B) West Java population (FLVQAGLRRRPTIGPRKRPAAS).

### Prediction of population coverage

**Fig. 9:**
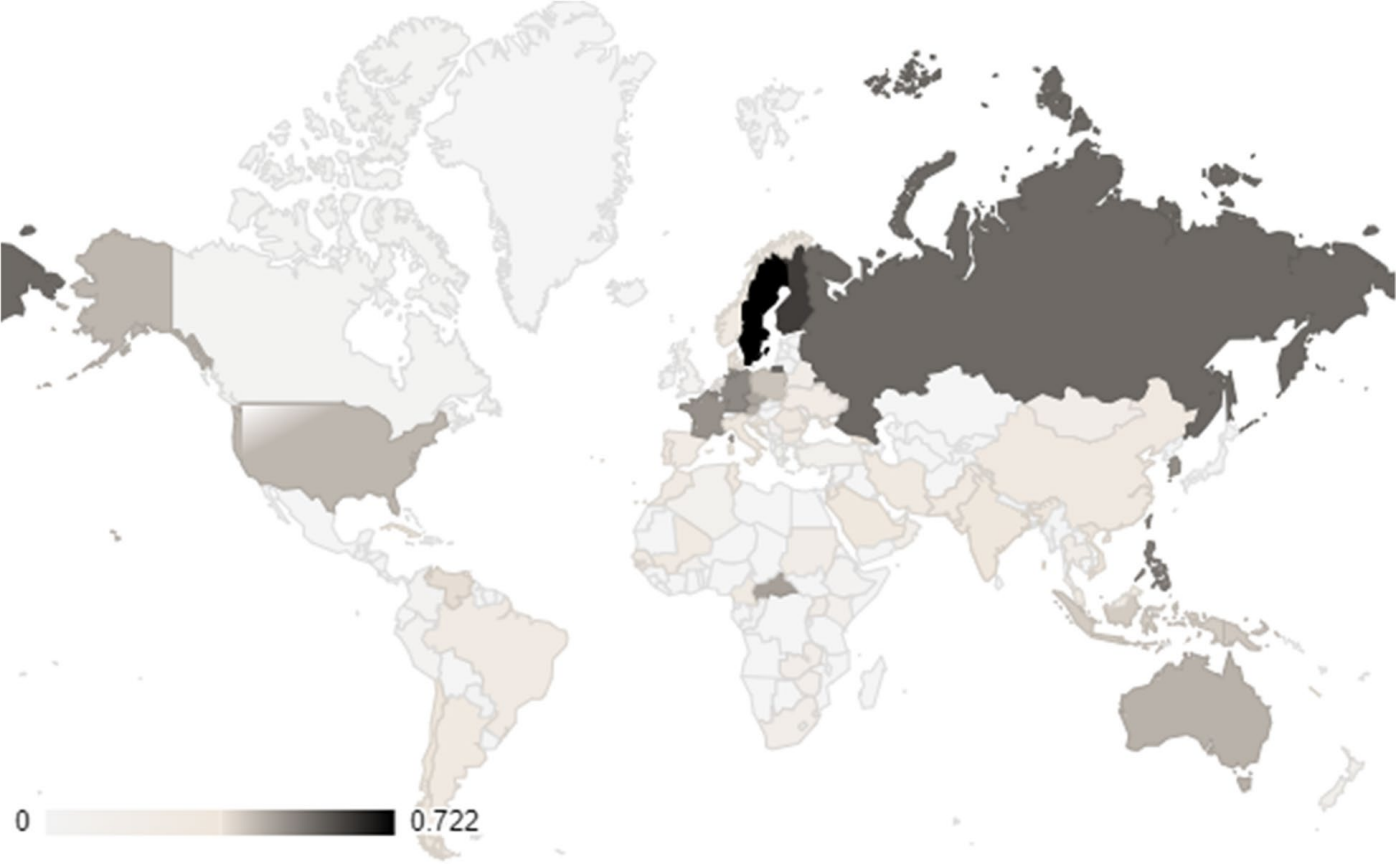
The predicted population coverage of the recombinant vaccine is based on the analysis of the selected HLA-1 and HLA-2 epitope data in combined form

The analysis result of the selected HLA-1 and HLA-2 epitope data in combined form is presented in Fig. 9. The population coverage data set as produced by the analysis is presented in Supplementary Table 6. The averaged data predicts that the designed recombinant protein will bind to 25.65% of the global population, with the lowest possibility being the population in Borneo, Fiji, Kiribati, Niue, and Paraguay (0.00%), and the highest being the population of Sweden (72.16%), whereas Indonesia, for whom the vaccine was designed, lies in the middle, with 40.18% population coverage.

## Discussion

Unlike immunoglobulins, which recognise and bind extracellular antigens and pathogens, the T cells recognise fragments of antigens presented on the surface of the host cells, before mediating a cascade of responses. These antigenic fragments are derived from the pathogen’s protein, or in vaccination from the vaccine protein, delivered and presented on the cell surface by the MHC, which in humans is often called HLA [13]. Thus, it is clear that for a vaccine to induce T cell responses, it must contain peptides that bind to HLA molecules, thus presented on the surface of antigen-presenting cells (APCs).

In addition to being polygenic, the MHC genes are also highly polymorphic, with high variations of every gene in the population [8]. Hence, it is imperative that for a vaccine to be protective of a certain group of people, it contains peptides recognised by the high frequency occurring HLA alleles in the population. Since the aim of our study was to create a protein vaccine against HPV suitable for the Indonesian population, we examined its compatibility with the high-frequency HLA alleles in the area [12], which are classified as HPV-related [9]. In addition, we also examined the protein against HPV-related high-frequency alleles in the general population [9–11].

Two highly antigenic peptides (EEYDLQFIF and KLKFWTVDLK) were recognised by high frequency HLA-1 alleles in both, the general (Table 3) and Western Javanese (Table 5), population. EEYDLQFIF attaches to both HLA-B*40 alleles present in the general (HLA-B*40:1) and Western Javanese (HLA-B*40:1 and HLA-B*40:2) population, whereas KLKFWTVDLK binds to HLA-A*03:01, which is present in both population groups. In addition to these two epitopes, there are a few more peptides recognised by the high-frequency Western Javanese HLA-1 alleles. These alleles, however, are not in Weiskopf’s list of high-frequency HLA-1 alleles in the general population.

Analysis of the highly antigenic epitopes binding to HLA-DRB1 alleles in both population groups indicated that although various length of peptides was found, only one peptide core was recognised by HLA-DRB1 alleles in each population. In general population, although three highly antigenic peptides with different lengths were found, they all have the same core and bind to the same antigen binding site (Table 4). The same is also true in the Western Javanese population, where 25 variations of highly antigenic peptides were found to have the same core and bind to the same antigen-binding site (Table 6). Both highly antigenic peptide cores associate with HLA-DRB1*04, although in the study of different population groups to different sub-types, due to the presence of different alleles in each population group. In addition to binding to HLA-DRB1*04:01 and HLA-DRB1*04:02, other HLA-DRB1*04 in the population, i.e., HLA-DRB1*04:03, HLA-DRB1*04:05, and HLA-DRB1*04:06 (Supplementary Table 5), also recognise certain peptides in the designed protein, albeit the lower antigenicity of these epitopes. These antigen-binding sites occur at high frequency in the Western Javanese population, although not in the general population.

This study proves that the designed protein contains highly antigenic epitopes (VaxiJen score >1), which are recognised by high-frequency HLA-1 and 2 alleles present in both the general and Western Javanese population. In addition, there are also some specific Western Javanese HLA alleles, which do not occur in the general population at such high frequency, that can recognise epitopes in the designed protein. Thus, it was expected that the protein is capable of eliciting T cell-mediated responses, both in the Western Javanese population and in the general population. However, when the population coverage of the epitopes binding HLA-1 and HLA-2 alleles was analysed, only nine countries, Germany, the UK (in this case only Northern Ireland and England, but not Wales nor Scotland), Belgium, Philippines, China (in this case Taiwan, but not the mainland nor Hong Kong), American Samoa, Russia, Finland, and Sweden, show more than 50% population coverage (Fig. 9 and Supplementary Table 6). The accuracy of this result is, of course, heavily influenced by the availability of data from each country. As can be observed in the allele distribution study (Figs. 2–5), no allele has been fully studied in all parts of the world; thus, bias is very likely to happen, conferring a seemingly higher level of protectiveness to areas with better-reported allele data compared to areas with fewer reported data, than it actually is. Thus, it has yet to be proven in vitro, whether or not the designed vaccine can confer suitable protection against HPV.

In the future, understanding the molecular genetic profiles of certain population groups will play even more important role in vaccine design and development, enabling targeted vaccination according to population molecular genetics in a more cost-effective manner. Since this study was solely based on in silico approach and the available population data to date, the consistency with in vivo data remains to be tested in the laboratory and in the field.

## Conclusion

The designed recombinant vaccine candidate based on protein L1 HPV45 used in this study is presumed to contain highly antigenic peptides that can bind HLA-1 and 2 alleles found at high frequency in general and Western Javanese populations. However, it has yet to be proven in vitro, whether or not the designed vaccine can confer suitable protection against HPV.

### Supplementary Information

**Additional file 1:** **Supplementary Table 1.** VaxiJen analysis of peptides with IEDB HLA-1 binding scores (to HPV-related high frequency alleles in general population) ≥ 0.6. Peptides with VaxiJen scores ≥1 were analysed further. Those in bold are antigenic peptides. **Supplementary Table 2.** VaxiJen analysis of peptides with IEDB HLA-2 binding scores (to HPV-related high frequency DQA1/DQB1 alleles in general population) ≥ 0.6. Peptides with VaxiJen scores ≥1 were analysed further. Those in bold are antigenic peptides. **Supplementary Table 3.** VaxiJen analysis of peptides with IEDB HLA-2 binding scores (to HPV-related high frequency DRB1 alleles in general population) ≥ 0.6. Peptides with VaxiJen scores ≥1 were analysed further. Those in bold are antigenic peptides. **Supplementary Table 4.** VaxiJen analysis of peptides with IEDB HLA-1 binding scores (to HPV-related high frequency HLA alleles in West Java) ≥ 0.6. Peptides with VaxiJen scores ≥1 were analysed further. Those in bold are antigenic peptides. **Supplementary Table 5.** VaxiJen analysis of peptides with IEDB HLA-2 adjusted percentile rank (upon binding to HPV-related high frequency HLA alleles in West Java) < 1. Peptides with VaxiJen scores ≥1 were analysed further. Those in bold are antigenic peptides. **Supplementary Table 6.** The population coverage data of the selected HLA-1 and HLA-2 epitope in combined form.

## Data Availability

HLA-1 and HLA-2 allele sets found at high frequency in the general population are available from https://help.iedb.org/hc/en-us/articles/114094151851. HLA-1 and HLA-2 allele sets found at high frequency in the Western Javanese population are available from 10.1111/j.1399-0039.2008.01178.x.

## Abbreviations

HLA: Human leukocyte antigen
HPV: Human papillomavirus
TLR: Toll-like receptors
MHC: Major histocompatibility complex
APCs: Antigen-presenting cells
