# Supplementary material for: In silico analysis of HLA-1 and HLA-2 recognition of a designed recombinant human papillomavirus vaccine based on L1 protein HPV subtype 45
Source: J Genet Eng Biotechnol. 2023 Dec 13;21:167. doi: 10.1186/s43141-023-00593-8 (PMC10719189; doi:10.1186/s43141-023-00593-8)
Supplement: Supplementary file 1 — Additional file 1: Supplementary Table 1. VaxiJen analysis of peptides with IEDB HLA-1 binding scores (to HPV-related high frequency alleles in general population) ≥ 0.6. Peptides with VaxiJen scores ≥1 were analysed further. Those in bold are antigenic peptides. Supplementary Table 2. VaxiJen analysis of peptides with IEDB HLA-2 binding scores (to HPV-related high frequency DQA1/DQB1 alleles in general population) ≥ 0.6. Peptides with VaxiJen scores ≥1 were analysed further. Those in bold are antigenic peptides. Supplementary Table 3. VaxiJen analysis of peptides with IEDB HLA-2 binding scores (to HPV-related high frequency DRB1 alleles in general population) ≥ 0.6. Peptides with VaxiJen scores ≥1 were analysed further. Those in bold are antigenic peptides. Supplementary Table 4. VaxiJen analysis of peptides with IEDB HLA-1 binding scores (to HPV-related high frequency HLA alleles in West Java) ≥ 0.6. Peptides with VaxiJen scores ≥1 were analysed further. Those in bold are antigenic peptides. Supplementary Table 5. VaxiJen analysis of peptides with IEDB HLA-2 adjusted percentile rank (upon binding to HPV-related high frequency HLA alleles in West Java) < 1. Peptides with VaxiJen scores ≥1 were analysed further. Those in bold are antigenic peptides. Supplementary Table 6. The population coverage data of the selected HLA-1 and HLA-2 epitope in combined form. [file 43141_2023_593_MOESM1_ESM.docx]

Supplementary Table 1 VaxiJen analysis of peptides with IEDB HLA-1 binding scores (to HPV-related high frequency alleles in general population) ≥ 0.6. Peptides with VaxiJen scores ≥1 were analysed further. Those in bold are antigenic peptides.

| **No** | **Peptide** | **Start** | **End** | **Length** | **Allele** | **VaxiJen Score** | **Antigen (A)/ Non-Antigen (NA)** |
| --- | --- | --- | --- | --- | --- | --- | --- |
| 1 | **EEYDLQFIF** | **398** | **406** | **9** | **HLA-B*40:01** | **1.7384** | **A** |
| 2 | **KLKFWTVDLK** | **473** | **482** | **10** | **HLA-A*03:01** | **1.1434** | **A** |
| 3 | **MEIGRGQPL** | **132** | **140** | **9** | **HLA-B*40:01** | **0.9861** | **A** |
| 4 | **TPPEKQDPY** | **463** | **471** | **9** | **HLA-B*35:01** | **0.9829** | **A** |
| 5 | **RPAKRVRI** | **525** | **532** | **8** | **HLA-B*07:02** | **0.9330** | **A** |
| 6 | **TQNPVPGTY** | **377** | **385** | **9** | **HLA-B*15:01** | **0.8973** | **A** |
| 7 | **RVVPSGAGNK** | **77** | **86** | **10** | **HLA-A*03:01** | **0.8424** | **A** |
| 8 | **STQNPVPGTY** | **376** | **385** | **10** | **HLA-B*15:01** | **0.7780** | **A** |
| 9 | **FPIFLQMAL** | **21** | **29** | **9** | **HLA-B*35:01** | **0.7776** | **A** |
|  |  |  |  |  | **HLA-B*07:02** |  |  |
| 10 | **SSILENWNF** | **426** | **434** | **9** | **HLA-B*58:01** | **0.7650** | **A** |
| 11 | **VPPPPTTSL** | **436** | **444** | **9** | **HLA-B*07:02** | **0.7072** | **A** |
|  |  |  |  |  | **HLA-B*35:01** |  |  |
| 12 | **ASTQNPVPGTY** | **375** | **385** | **11** | **HLA-B*15:01** | **0.7019** | **A** |
| 13 | **GVPPPPTTSL** | **435** | **444** | **10** | **HLA-B*07:02** | **0.6223** | **A** |
| 14 | **KAQGHNNGICW** | **344** | **354** | **11** | **HLA-B*58:01** | **0.5989** | **A** |
| 15 | **FGVPPPPTTSL** | **434** | **444** | **11** | **HLA-B*07:02** | **0.5527** | **A** |
| 16 | **GLSGHPFYNK** | **143** | **152** | **10** | **HLA-A*03:01** | **0.5524** | **A** |
| 17 | **RLLTVGNPY** | **67** | **75** | **9** | **HLA-B*15:01** | **0.5385** | **A** |
| 18 | **GTYDPTKFK** | **383** | **391** | **9** | **HLA-A*03:01** | **0.4814** | **A** |
| 19 | **VPGTYDPTKFK** | **381** | **391** | **11** | **HLA-A*03:01** | **0.4638** | **A** |
| 20 | **YLPPPSVARV** | **38** | **47** | **10** | **HLA-A*02:03** | **0.4104** | **A** |
|  |  |  |  |  | **HLA-A*02:01** |  |  |
|  |  |  |  |  | **HLA-A*02:06** |  |  |
| 21 | YVSRTSIFY | 53 | 61 | 9 | HLA-B*35:01 | 0.3935 | NA |
|  |  |  |  |  | HLA-B*15:01 |  |  |
| 22 | TLTAEVMSY | 412 | 420 | 9 | HLA-B*15:01 | 0.3720 | NA |
| 23 | LPDPNKFGL | 104 | 112 | 9 | HLA-B*07:02 | 0.3282 | NA |
|  |  |  |  |  | HLA-B*35:01 |  |  |
| 24 | QAVPKVSAY | 87 | 95 | 9 | HLA-B*35:01 | 0.3232 | NA |
|  |  |  |  |  | HLA-B*15:01 |  |  |
| 25 | GEHWAKGTL | 193 | 201 | 9 | HLA-B*40:01 | 0.3085 | NA |
| 26 | SPSPSGSI | 321 | 328 | 8 | HLA-B*07:02 | 0.2827 | NA |
| 27 | QLFNKPYWLHK | 334 | 344 | 11 | HLA-A*03:01 | 0.2168 | NA |
| 28 | FGLPDSTIY | 110 | 118 | 9 | HLA-B*35:01 | 0.1881 | NA |
| 29 | QLFNKPYWL | 334 | 342 | 9 | HLA-A*02:01 | 0.1842 | NA |
| 30 | TVYLPPPSV | 36 | 44 | 9 | HLA-A*02:06 | 0.1695 | NA |
|  |  |  |  |  | HLA-A*02:03 |  |  |
|  |  |  |  |  | HLA-A*02:01 |  |  |
| 31 | RPAASTSTA | 515 | 523 | 9 | HLA-B*07:02 | 0.1505 | NA |
| 32 | RPSDSTVYL | 31 | 39 | 9 | HLA-B*07:02 | 0.1266 | NA |
|  |  |  |  |  | HLA-B*35:01 |  |  |
| 33 | RVALPDPNK | 101 | 109 | 9 | HLA-A*03:01 | 0.0447 | NA |
| 34 | ITTSDSQLF | 328 | 336 | 9 | HLA-B*58:01 | 0.0385 | NA |
| 35 | TSLVDTYRF | 442 | 450 | 9 | HLA-B*58:01 | 0.0321 | NA |
| 36 | SLVDTYRFV | 443 | 451 | 9 | HLA-A*02:03 | 0.0137 | NA |
|  |  |  |  |  | HLA-A*02:01 |  |  |
|  |  |  |  |  | HLA-A*02:06 |  |  |
| 37 | AEVMSYIHSM | 415 | 424 | 10 | HLA-B*40:01 | -0.0021 | NA |
| 38 | VPKVSAYQY | 89 | 97 | 9 | HLA-B*35:01 | -0.0555 | NA |
| 39 | EVMSYIHSM | 416 | 424 | 9 | HLA-B*35:01 | -0.0795 | NA |
| 40 | HSMNSSILENW | 422 | 432 | 11 | HLA-B*58:01 | -0.1575 | NA |
| 41 | RVALPDPNKF | 101 | 110 | 10 | HLA-B*58:01 | -0.2093 | NA |
| 42 | KQAVPKVSAY | 86 | 95 | 10 | HLA-B*15:01 | -0.2162 | NA |
| 43 | DPYGDSMFF | 267 | 275 | 9 | HLA-B*35:01 | -0.2414 | NA |
| 44 | VALPDPNKF | 102 | 110 | 9 | HLA-B*58:01 | -0.2866 | NA |
|  |  |  |  |  | HLA-B*35:01 |  |  |
| 45 | MNSSILENW | 424 | 432 | 9 | HLA-B*58:01 | -0.2954 | NA |
| 46 | TIYNPETQR | 116 | 124 | 9 | HLA-A*03:01 | -0.3554 | NA |
| 47 | ALWRPSDSTVY | 28 | 38 | 11 | HLA-B*15:01 | -0.4170 | NA |
| 48 | LDQYPLGRKF | 489 | 498 | 10 | HLA-B*15:01 | -0.4185 | NA |
| 49 | GCVPAIGEHW | 187 | 196 | 10 | HLA-B*58:01 | -0.5060 | NA |

Supplementary Table 2 VaxiJen analysis of peptides with IEDB HLA-2 binding scores (to HPV-related high frequency DQA1/DQB1 alleles in general population) ≥ 0.6. Peptides with VaxiJen scores ≥1 were analysed further. Those in bold are antigenic peptides.

| **No** | **Peptide** | **Start** | **End** | **Length** | **Allele** | **VaxiJen Score** | **Antigenic/**  **Non-Antigenic** | **netmhciipan_core** | **netmhciipan_ic50** |
| --- | --- | --- | --- | --- | --- | --- | --- | --- | --- |
| 1 | **KLDDTESAHAATAVI** | **152** | **166** | **15** | **HLA-DQA1*03:01/DQB1*03:01** | **0.6551** | **A** | TESAHAATA | 60.26 |
| 2 | **KLDDTESAHAATAVIT** | **152** | **167** | **16** | **HLA-DQA1*03:01/DQB1*03:01** | **0.6504** | **A** | TESAHAATA | 38.98 |
|  |  |  |  |  | **HLA-DQA1*01:02/DQB1*03:01** |  |  | TESAHAATA | 69.12 |
| 3 | **KLDDTESAHAATAVITQ** | **152** | **168** | **17** | **HLA-DQA1*03:01/DQB1*03:01** | **0.6162** | **A** | AHAATAVIT | 41.52 |
|  |  |  |  |  | **HLA-DQA1*01:02/DQB1*03:01** |  |  | AHAATAVIT | 65.78 |
| 4 | **LDDTESAHAATAVI** | **153** | **166** | **14** | **HLA-DQA1*03:01/DQB1*03:01** | **0.6084** | **A** | TESAHAATA | 74.97 |
| 5 | **LDDTESAHAATAVIT** | **153** | **167** | **15** | **HLA-DQA1*03:01/DQB1*03:01** | **0.6061** | **A** | AHAATAVIT | 37.69 |
|  |  |  |  |  | **HLA-DQA1*01:02/DQB1*03:01** |  |  | TESAHAATA | 73.02 |
| 6 | **TESAHAATAVITQDVRD** | **156** | **172** | **17** | **HLA-DQA1*03:01/DQB1*03:01** | **0.6009** | **A** | AHAATAVIT | 46.87 |
|  |  |  |  |  | **HLA-DQA1*01:02/DQB1*03:01** |  |  | AHAATAVIT | 71.98 |
| 7 | **LDDTESAHAATAVITQ** | **153** | **168** | **16** | **HLA-DQA1*03:01/DQB1*03:01** | **0.5727** | **A** | AHAATAVIT | 36.98 |
|  |  |  |  |  | **HLA-DQA1*01:02/DQB1*03:01** |  |  | AHAATAVIT | 63.21 |
|  |  |  |  |  | **HLA-DQA1*03:01/DQB1*06:02** |  |  | TESAHAATA | 272.26 |
| 8 | **ESAHAATAVITQDVRD** | **157** | **172** | **16** | **HLA-DQA1*03:01/DQB1*03:01** | **0.5725** | **A** | AHAATAVIT | 51.82 |
|  |  |  |  |  | **HLA-DQA1*01:02/DQB1*03:01** |  |  | AHAATAVIT | 88.22 |
| 9 | **NKLDDTESAHAATAVIT** | **151** | **167** | **17** | **HLA-DQA1*03:01/DQB1*03:01** | **0.5617** | **A** | TESAHAATA | 43.43 |
|  |  |  |  |  | **HLA-DQA1*01:02/DQB1*03:01** |  |  | TESAHAATA | 72.29 |
| 10 | **DTESAHAATAVITQDVR** | **155** | **171** | **17** | **HLA-DQA1*03:01/DQB1*03:01** | **0.5536** | **A** | AHAATAVIT | 41.73 |
|  |  |  |  |  | **HLA-DQA1*01:02/DQB1*03:01** |  |  | AHAATAVIT | 64.54 |
| 11 | **TESAHAATAVITQDVR** | **156** | **171** | **16** | **HLA-DQA1*03:01/DQB1*03:01** | **0.5471** | **A** | AHAATAVIT | 39.84 |
|  |  |  |  |  | **HLA-DQA1*01:02/DQB1*03:01** |  |  | AHAATAVIT | 65.41 |
|  |  |  |  |  | **HLA-DQA1*01:02/DQB1*03:02** |  |  | AATAVITQD | 483.01 |
|  |  |  |  |  | **HLA-DQA1*03:01/DQB1*06:02** |  |  | AHAATAVIT | 281.2 |
| 12 | **SAHAATAVITQDVRD** | **158** | **172** | **15** | **HLA-DQA1*03:01/DQB1*03:01** | **0.5411** | **A** | AHAATAVIT | 59.04 |
| 13 | **LDDTESAHAATAVITQD** | **153** | **169** | **17** | **HLA-DQA1*03:01/DQB1*03:01** | **0.5162** | **A** | AHAATAVIT | 40.82 |
|  |  |  |  |  | **HLA-DQA1*01:02/DQB1*03:01** |  |  | AHAATAVIT | 64.83 |
| 14 | **ESAHAATAVITQDVR** | **157** | **171** | **15** | **HLA-DQA1*03:01/DQB1*03:01** | **0.5124** | **A** | AHAATAVIT | 45.36 |
|  |  |  |  |  | **HLA-DQA1*01:02/DQB1*03:01** |  |  | AHAATAVIT | 83.19 |
|  |  |  |  |  | **HLA-DQA1*01:02/DQB1*03:02** |  |  | AATAVITQD | 505.6 |
| 15 | **SAHAATAVITQDVR** | **158** | **171** | **14** | **HLA-DQA1*03:01/DQB1*03:01** | **0.4726** | **A** | AHAATAVIT | 60.83 |
|  |  |  |  |  | **HLA-DQA1*01:02/DQB1*03:01** |  |  | AHAATAVIT | 120.58 |
|  |  |  |  |  | **HLA-DQA1*01:02/DQB1*03:02** |  |  | AATAVITQD | 571.22 |
| 16 | **DTESAHAATAVIT** | **155** | **167** | **13** | **HLA-DQA1*03:01/DQB1*03:01** | **0.4535** | **A** | AHAATAVIT | 56.9 |
|  |  |  |  |  | **HLA-DQA1*01:02/DQB1*03:01** |  |  | AHAATAVIT | 122.89 |
| 17 | **DDTESAHAATAVIT** | **154** | **167** | **14** | **HLA-DQA1*03:01/DQB1*03:01** | **0.4517** | **A** | AHAATAVIT | 45.57 |
|  |  |  |  |  | **HLA-DQA1*01:02/DQB1*03:01** |  |  | AHAATAVIT | 95.38 |
| 18 | **LDDTESAHAATAVITQDV** | **153** | **170** | **18** | **HLA-DQA1*03:01/DQB1*03:01** | **0.4375** | **A** | AHAATAVIT | 42.24 |
| 19 | **DTESAHAATAVITQ** | **155** | **168** | **14** | **HLA-DQA1*03:01/DQB1*03:01** | **0.4288** | **A** | AHAATAVIT | 40.46 |
|  |  |  |  |  | **HLA-DQA1*01:02/DQB1*03:01** |  |  | AHAATAVIT | 81.85 |
|  |  |  |  |  | **HLA-DQA1*03:01/DQB1*06:02** |  |  | AHAATAVIT | 395.09 |
| 20 | **DDTESAHAATAVITQ** | **154** | **168** | **15** | **HLA-DQA1*03:01/DQB1*03:01** | **0.4282** | **A** | AHAATAVIT | 35.97 |
|  |  |  |  |  | **HLA-DQA1*01:02/DQB1*03:01** |  |  | AHAATAVIT | 68.07 |
|  |  |  |  |  | **HLA-DQA1*03:01/DQB1*06:02** |  |  | AHAATAVIT | 326.09 |
| 21 | **TESAHAATAVIT** | **156** | **167** | **12** | **HLA-DQA1*01:02/DQB1*03:01** | **0.4269** | **A** | AHAATAVIT | 166.6 |
|  |  |  |  |  | **HLA-DQA1*03:01/DQB1*03:01** |  |  | AHAATAVIT | 72.89 |
| 22 | **TESAHAATAVITQ** | **156** | **168** | **13** | **HLA-DQA1*01:02/DQB1*03:01** | **0.4048** | **A** | AHAATAVIT | 111.08 |
|  |  |  |  |  | **HLA-DQA1*03:01/DQB1*03:01** |  |  | AHAATAVIT | 53.05 |
| 23 | DDTESAHAATAVITQD | 154 | 169 | 16 | HLA-DQA1*03:01/DQB1*03:01 | 0.3781 | NA | AHAATAVIT | 37.91 |
|  |  |  |  |  | HLA-DQA1*01:02/DQB1*03:01 |  |  | AHAATAVIT | 65.23 |
| 24 | DTESAHAATAVITQD | 155 | 169 | 15 | HLA-DQA1*03:01/DQB1*03:01 | 0.3751 | NA | AHAATAVIT | 36.51 |
|  |  |  |  |  | HLA-DQA1*01:02/DQB1*03:01 |  |  | AHAATAVIT | 67.08 |
|  |  |  |  |  | HLA-DQA1*03:01/DQB1*06:02 |  |  | AHAATAVIT | 309.46 |
| 25 | TESAHAATAVITQD | 156 | 169 | 14 | HLA-DQA1*03:01/DQB1*03:01 | 0.3498 | NA | AHAATAVIT | 43.01 |
|  |  |  |  |  | HLA-DQA1*01:02/DQB1*03:01 |  |  | AHAATAVIT | 85.26 |
|  |  |  |  |  | HLA-DQA1*01:02/DQB1*03:02 |  |  | AATAVITQD | 598.91 |
|  |  |  |  |  | HLA-DQA1*03:01/DQB1*06:02 |  |  | AHAATAVIT | 404.74 |
| 26 | ESAHAATAVITQ | 157 | 168 | 12 | HLA-DQA1*03:01/DQB1*03:01 | 0.3439 | NA | AHAATAVIT | 80.36 |
|  |  |  |  |  | HLA-DQA1*01:02/DQB1*03:01 |  |  | AHAATAVIT | 184.05 |
| 27 | DDTESAHAATAVITQDV | 154 | 170 | 17 | HLA-DQA1*03:01/DQB1*03:01 | 0.3026 | NA | AHAATAVIT | 40.56 |
|  |  |  |  |  | HLA-DQA1*01:02/DQB1*03:01 |  |  | AHAATAVIT | 62.84 |
| 28 | DTESAHAATAVITQDV | 155 | 170 | 16 | HLA-DQA1*03:01/DQB1*03:01 | 0.2937 | NA | AHAATAVIT | 36.65 |
|  |  |  |  |  | HLA-DQA1*01:02/DQB1*03:01 |  |  | AHAATAVIT | 60.34 |
|  |  |  |  |  | HLA-DQA1*01:02/DQB1*03:02 |  |  | AATAVITQD | 454.7 |
|  |  |  |  |  | HLA-DQA1*03:01/DQB1*06:02 |  |  | AHAATAVIT | 267.67 |
| 29 | ESAHAATAVITQD | 157 | 169 | 13 | HLA-DQA1*03:01/DQB1*03:01 | 0.2895 | NA | AHAATAVIT | 67.51 |
|  |  |  |  |  | HLA-DQA1*01:02/DQB1*03:01 |  |  | AHAATAVIT | 140.5 |
| 30 | TESAHAATAVITQDV | 156 | 170 | 15 | HLA-DQA1*03:01/DQB1*03:01 | 0.2637 | NA | AHAATAVIT | 36.81 |
|  |  |  |  |  | HLA-DQA1*01:02/DQB1*03:01 |  |  | AHAATAVIT | 64.57 |
|  |  |  |  |  | HLA-DQA1*01:02/DQB1*03:02 |  |  | AATAVITQD | 438.87 |
|  |  |  |  |  | HLA-DQA1*03:01/DQB1*06:02 |  |  | AHAATAVIT | 295.21 |
| 31 | SAHAATAVITQD | 158 | 169 | 12 | HLA-DQA1*01:02/DQB1*03:01 | 0.2142 | NA | AHAATAVIT | 211.52 |
|  |  |  |  |  | HLA-DQA1*03:01/DQB1*03:01 |  |  | AHAATAVIT | 100.69 |
| 32 | ESAHAATAVITQDV | 157 | 170 | 14 | HLA-DQA1*03:01/DQB1*03:01 | 0.1997 | NA | AHAATAVIT | 49.55 |
|  |  |  |  |  | HLA-DQA1*01:02/DQB1*03:01 |  |  | AHAATAVIT | 95.82 |
|  |  |  |  |  | HLA-DQA1*01:02/DQB1*03:02 |  |  | AATAVITQD | 498.83 |
| 33 | SIFYHAGSSRLLTVG | 58 | 72 | 15 | HLA-DQA1*03:01/DQB1*04:02 | 0.1451 | NA | YHAGSSRLL | 588.86 |
| 34 | SAHAATAVITQDV | 158 | 170 | 13 | HLA-DQA1*01:02/DQB1*03:02 | 0.1226 | NA | AATAVITQD | 605.89 |
|  |  |  |  |  | HLA-DQA1*01:02/DQB1*03:01 |  |  | AHAATAVIT | 143.07 |
|  |  |  |  |  | HLA-DQA1*03:01/DQB1*03:01 |  |  | AHAATAVIT | 73.81 |
| 35 | PKVSAYQYRVFRVA | 90 | 103 | 14 | HLA-DQA1*03:01/DQB1*04:02 | 0.0989 | NA | AYQYRVFRV | 601.42 |
| 36 | AHAATAVITQDV | 159 | 170 | 12 | HLA-DQA1*01:02/DQB1*03:02 | 0.0814 | NA | AATAVITQD | 839.46 |
| 37 | KVSAYQYRVFRVA | 91 | 103 | 13 | HLA-DQA1*03:01/DQB1*04:02 | 0.0779 | NA | AYQYRVFRV | 635.27 |
| 38 | PKVSAYQYRVFRVAL | 90 | 104 | 15 | HLA-DQA1*03:01/DQB1*04:02 | 0.0363 | NA | AYQYRVFRV | 537.13 |
| 39 | KVSAYQYRVFRVAL | 91 | 104 | 14 | HLA-DQA1*03:01/DQB1*04:02 | 0.0107 | NA | AYQYRVFRV | 513.25 |
| 40 | VSAYQYRVFRVALPDPN | 92 | 108 | 17 | HLA-DQA1*03:01/DQB1*04:02 | 0.0093 | NA | YRVFRVALP | 516.82 |
| 41 | VSAYQYRVFRVAL | 92 | 104 | 13 | HLA-DQA1*03:01/DQB1*04:02 | 0.0022 | NA | AYQYRVFRV | 634.8 |
| 42 | PKVSAYQYRVFRVALPD | 90 | 106 | 17 | HLA-DQA1*03:01/DQB1*04:02 | -0.0051 | NA | YRVFRVALP | 517.35 |
| 43 | KVSAYQYRVFRVALPD | 91 | 106 | 16 | HLA-DQA1*03:01/DQB1*04:02 | -0.0276 | NA | YRVFRVALP | 434.47 |
| 44 | SAYQYRVFRVALPDPN | 93 | 108 | 16 | HLA-DQA1*03:01/DQB1*04:02 | -0.035 | NA | YRVFRVALP | 465.09 |
|  |  |  |  |  | HLA-DQA1*03:01/DQB1*05:01 |  |  | YRVFRVALP | 288.82 |
| 45 | VSAYQYRVFRVALPD | 92 | 106 | 15 | HLA-DQA1*03:01/DQB1*04:02 | -0.0397 | NA | YRVFRVALP | 391.75 |
| 46 | SAYQYRVFRVAL | 93 | 104 | 12 | HLA-DQA1*03:01/DQB1*04:02 | -0.058 | NA | AYQYRVFRV | 765.05 |
| 47 | KVSAYQYRVFRVALPDP | 91 | 107 | 17 | HLA-DQA1*03:01/DQB1*04:02 | -0.0605 | NA | YRVFRVALP | 504.38 |
| 48 | AYQYRVFRVALPDPN | 94 | 108 | 15 | HLA-DQA1*03:01/DQB1*04:02 | -0.0707 | NA | YRVFRVALP | 462.92 |
|  |  |  |  |  | HLA-DQA1*03:01/DQB1*05:01 |  |  | YRVFRVALP | 309.06 |
| 49 | VSAYQYRVFRVALPDP | 92 | 107 | 16 | HLA-DQA1*03:01/DQB1*04:02 | -0.0766 | NA | YRVFRVALP | 457.04 |
|  |  |  |  |  | HLA-DQA1*03:01/DQB1*05:01 |  |  | YRVFRVALP | 299.62 |
| 50 | RTSIFYHAGSSRLLTV | 56 | 71 | 16 | HLA-DQA1*03:01/DQB1*04:02 | -0.0896 | NA | FYHAGSSRL | 557.3 |
| 51 | SAYQYRVFRVALPD | 93 | 106 | 14 | HLA-DQA1*03:01/DQB1*04:02 | -0.0935 | NA | YRVFRVALP | 401.21 |
|  |  |  |  |  | HLA-DQA1*03:01/DQB1*05:01 |  |  | YRVFRVALP | 342.49 |
| 52 | PKVSAYQYRVFRVALP | 90 | 105 | 16 | HLA-DQA1*03:01/DQB1*04:02 | -0.1018 | NA | AYQYRVFRV | 438.06 |
| 53 | SRTSIFYHAGSSRLL | 55 | 69 | 15 | HLA-DQA1*03:01/DQB1*04:02 | -0.1267 | NA | FYHAGSSRL | 567.45 |
| 54 | SAYQYRVFRVALPDP | 93 | 107 | 15 | HLA-DQA1*03:01/DQB1*04:02 | -0.1315 | NA | YRVFRVALP | 412.46 |
|  |  |  |  |  | HLA-DQA1*03:01/DQB1*05:01 |  |  | YRVFRVALP | 292.8 |
| 55 | KVSAYQYRVFRVALP | 91 | 105 | 15 | HLA-DQA1*03:01/DQB1*04:02 | -0.1363 | NA | AYQYRVFRV | 369.96 |
| 56 | AYQYRVFRVALPD | 94 | 106 | 13 | HLA-DQA1*03:01/DQB1*04:02 | -0.1422 | NA | YRVFRVALP | 503.34 |
| 57 | SAYQYRVFRVALPDPNK | 93 | 109 | 17 | HLA-DQA1*03:01/DQB1*04:02 | -0.1478 | NA | YRVFRVALP | 537.35 |
| 58 | VSAYQYRVFRVALP | 92 | 105 | 14 | HLA-DQA1*03:01/DQB1*04:02 | -0.1597 | NA | YRVFRVALP | 391.28 |
|  |  |  |  |  | HLA-DQA1*03:01/DQB1*05:01 |  |  | AYQYRVFRV | 343.32 |
| 59 | TSIFYHAGSSRLLTV | 57 | 71 | 15 | HLA-DQA1*03:01/DQB1*04:02 | -0.18 | NA | YHAGSSRLL | 531.57 |
| 60 | AYQYRVFRVALPDP | 94 | 107 | 14 | HLA-DQA1*03:01/DQB1*04:02 | -0.1805 | NA | YRVFRVALP | 471.71 |
| 61 | SRTSIFYHAGSSRLLT | 55 | 70 | 16 | HLA-DQA1*03:01/DQB1*04:02 | -0.1848 | NA | FYHAGSSRL | 578.49 |
| 62 | AYQYRVFRVALPDPNK | 94 | 109 | 16 | HLA-DQA1*03:01/DQB1*04:02 | -0.1879 | NA | YRVFRVALP | 526.69 |
| 63 | VPKVSAYQYRVFRVALP | 89 | 105 | 17 | HLA-DQA1*03:01/DQB1*04:02 | -0.2128 | NA | AYQYRVFRV | 512.03 |
| 64 | SAYQYRVFRVALP | 93 | 105 | 13 | HLA-DQA1*03:01/DQB1*04:02 | -0.232 | NA | YRVFRVALP | 434.28 |
| 65 | SIFYHAGSSRLLTV | 58 | 71 | 14 | HLA-DQA1*03:01/DQB1*04:02 | -0.2624 | NA | YHAGSSRLL | 587.77 |
| 66 | RTSIFYHAGSSRLLT | 56 | 70 | 15 | HLA-DQA1*03:01/DQB1*04:02 | -0.2758 | NA | YHAGSSRLL | 568.11 |
| 67 | AYQYRVFRVALP | 94 | 105 | 12 | HLA-DQA1*03:01/DQB1*04:02 | -0.3062 | NA | YRVFRVALP | 569.61 |
| 68 | QLFARHFWNRAGVM | 281 | 294 | 14 | HLA-DQA1*03:01/DQB1*04:02 | -0.3142 | NA | FARHFWNRA | 608.9 |
| 69 | EQLFARHFWNRAGVM | 280 | 294 | 15 | HLA-DQA1*03:01/DQB1*04:02 | -0.3544 | NA | FARHFWNRA | 604.92 |
| 70 | TSIFYHAGSSRLLT | 57 | 70 | 14 | HLA-DQA1*03:01/DQB1*04:02 | -0.3906 | NA | YHAGSSRLL | 639 |

Supplementary Table 3 VaxiJen analysis of peptides with IEDB HLA-2 binding scores (to HPV-related high frequency DRB1 alleles in general population) ≥ 0.6. Peptides with VaxiJen scores ≥1 were analysed further. Those in bold are antigenic peptides.

| **No** | **Peptide** | **Start** | **End** | **Length** | **Allele** | **VaxiJen Score** | **Antigenic**  **/ Non Antigenic** |
| --- | --- | --- | --- | --- | --- | --- | --- |
| 1 | **DLYIKGTSANMRET** | **301** | **314** | **14** | **HLA-DRB1*04:01** | **1.104** | **A** |
| 2 | **DLYIKGTSANMRETP** | **301** | **315** | **15** | **HLA-DRB1*04:01** | **1.091** | **A** |
| 3 | **TDLYIKGTSANMRET** | **300** | **314** | **15** | **HLA-DRB1*04:01** | **1.0182** | **A** |
| 4 | **DLYIKGTSANMRE** | **301** | **313** | **13** | **HLA-DRB1*04:01** | **0.9763** | **A** |
| 5 | **GNPYFRVVPSGAG** | **72** | **84** | **13** | **HLA-DRB1*09:01** | **0.9729** | **A** |
| 6 | **TVGNPYFRVVPSGAG** | **70** | **84** | **15** | **HLA-DRB1*09:01** | **0.9302** | **A** |
| 7 | **VGNPYFRVVPSGAG** | **71** | **84** | **14** | **HLA-DRB1*09:01** | **0.9195** | **A** |
| 8 | **TDLYIKGTSANMRE** | **300** | **313** | **14** | **HLA-DRB1*04:01** | **0.8955** | **A** |
| 9 | **GNPYFRVVPSGAGNK** | **72** | **86** | **15** | **HLA-DRB1*09:01** | **0.8915** | **A** |
| 10 | **NPYFRVVPSGAGNK** | **73** | **86** | **14** | **HLA-DRB1*09:01** | **0.8789** | **A** |
| 11 | **IIIFLKNVNVFPI** | **11** | **23** | **13** | **HLA-DRB1*13:02** | **0.8701** | **A** |
|  |  | **11** | **23** |  | **HLA-DRB1*15:01** | **0.8701** | **A** |
| 12 | **GNPYFRVVPSGAGN** | **72** | **85** | **14** | **HLA-DRB1*09:01** | **0.8452** | **A** |
| 13 | **GIIIFLKNVNVFPI** | **10** | **23** | **14** | **HLA-DRB1*15:01** | **0.8396** | **A** |
|  |  | **10** | **23** |  | **HLA-DRB1*13:02** | **0.8396** | **A** |
| 14 | **PTDLYIKGTSANMRE** | **299** | **313** | **15** | **HLA-DRB1*04:01** | **0.8283** | **A** |
| 15 | **NPYFRVVPSGAGN** | **73** | **85** | **13** | **HLA-DRB1*09:01** | **0.8211** | **A** |
| 16 | **IIFLKNVNVFPIF** | **12** | **24** | **13** | **HLA-DRB1*13:02** | **0.8128** | **A** |
| 17 | **VGNPYFRVVPSGAGN** | **71** | **85** | **15** | **HLA-DRB1*09:01** | **0.8093** | **A** |
| 18 | **IIFLKNVNVFPIFLQ** | **12** | **26** | **15** | **HLA-DRB1*13:02** | **0.7925** | **A** |
| 19 | **IIFLKNVNVFPIFL** | **12** | **25** | **14** | **HLA-DRB1*13:02** | **0.7787** | **A** |
| 20 | **IIIFLKNVNVFPIFLQM** | **11** | **27** | **17** | **HLA-DRB1*15:01** | **0.7552** | **A** |
| 21 | **GIIIFLKNVNVFPIFLQM** | **10** | **27** | **18** | **HLA-DRB1*15:01** | **0.7477** | **A** |
| 22 | **IIIFLKNVNVFPIF** | **11** | **24** | **14** | **HLA-DRB1*13:02** | **0.7318** | **A** |
|  |  | **11** | **24** |  | **HLA-DRB1*15:01** | **0.7318** | **A** |
| 23 | **IIIFLKNVNVFPIFLQ** | **11** | **26** | **16** | **HLA-DRB1*15:01** | **0.7254** | **A** |
|  |  | **11** | **26** |  | **HLA-DRB1*13:02** | **0.7254** | **A** |
| 24 | **GIIIFLKNVNVFPIFLQ** | **10** | **26** | **17** | **HLA-DRB1*15:01** | **0.7176** | **A** |
| 25 | **GIIIFLKNVNVFPIF** | **10** | **24** | **15** | **HLA-DRB1*15:01** | **0.7168** | **A** |
|  |  | **10** | **24** |  | **HLA-DRB1*13:02** | **0.7168** | **A** |
| 26 | **IIIFLKNVNVFPIFL** | **11** | **25** | **15** | **HLA-DRB1*13:02** | **0.7079** | **A** |
|  |  | **11** | **25** |  | **HLA-DRB1*15:01** | **0.7079** | **A** |
| 27 | **GIIIFLKNVNVFPIFL** | **10** | **25** | **16** | **HLA-DRB1*15:01** | **0.6992** | **A** |
|  |  | **10** | **25** |  | **HLA-DRB1*13:02** | **0.6992** | **A** |
| 28 | **GIIIFLKNVNVFP** | **10** | **22** | **13** | **HLA-DRB1*15:01** | **0.696** | **A** |
| 29 | **HGIIIFLKNVNVFPI** | **9** | **23** | **15** | **HLA-DRB1*15:01** | **0.6794** | **A** |
|  |  | **9** | **23** |  | **HLA-DRB1*13:02** | **0.6794** | **A** |
| 30 | **HGIIIFLKNVNV** | **9** | **20** | **12** | **HLA-DRB1*15:01** | **0.6544** | **A** |
| 31 | **HGIIIFLKNVNVFPIFLQM** | **9** | **27** | **19** | **HLA-DRB1*15:01** | **0.6289** | **A** |
| 32 | **HGIIIFLKNVNVFPIFLQ** | **9** | **26** | **18** | **HLA-DRB1*15:01** | **0.5935** | **A** |
| 33 | **HGIIIFLKNVNVFPIF** | **9** | **24** | **16** | **HLA-DRB1*15:01** | **0.5756** | **A** |
|  |  | **9** | **24** |  | **HLA-DRB1*13:02** | **0.5756** | **A** |
| 34 | **MSYIHSMNSSILENWNF** | **418** | **434** | **17** | **HLA-DRB1*04:05** | **0.5693** | **A** |
| 35 | **HGIIIFLKNVNVFPIFL** | **9** | **25** | **17** | **HLA-DRB1*15:01** | **0.5688** | **A** |
| 36 | **HGIIIFLKNVNVFP** | **9** | **22** | **14** | **HLA-DRB1*15:01** | **0.535** | **A** |
| 37 | **GHGIIIFLKNVNVFPI** | **8** | **23** | **16** | **HLA-DRB1*15:01** | **0.5293** | **A** |
|  |  | **8** | **23** |  | **HLA-DRB1*13:02** | **0.5293** | **A** |
| 38 | **YGHGIIIFLKNVNVFPI** | **7** | **23** | **17** | **HLA-DRB1*15:01** | **0.5122** | **A** |
| 39 | **GHGIIIFLKNVNVFPIFLQ** | **8** | **26** | **19** | **HLA-DRB1*15:01** | **0.4788** | **A** |
| 40 | **SYIHSMNSSILENWNF** | **419** | **434** | **16** | **HLA-DRB1*09:01** | **0.471** | **A** |
| 41 | **GHGIIIFLKNVNV** | **8** | **20** | **13** | **HLA-DRB1*15:01** | **0.4579** | **A** |
| 42 | **GHGIIIFLKNVNVFPIFL** | **8** | **25** | **18** | **HLA-DRB1*15:01** | **0.4476** | **A** |
| 43 | **YGHGIIIFLKNVNV** | **7** | **20** | **14** | **HLA-DRB1*15:01** | **0.4445** | **A** |
| 44 | **GHGIIIFLKNVNVFPIF** | **8** | **24** | **17** | **HLA-DRB1*15:01** | **0.4438** | **A** |
| 45 | **YGHGIIIFLKNVNVFPIFL** | **7** | **25** | **19** | **HLA-DRB1*15:01** | **0.4376** | **A** |
| 46 | **YGHGIIIFLKNVNVFPIF** | **7** | **24** | **18** | **HLA-DRB1*15:01** | **0.4327** | **A** |
| 47 | **FYHAGSSRLLTVGNPYFR** | **60** | **77** | **18** | **HLA-DRB1*09:01** | **0.4208** | **A** |
| 48 | **FYHAGSSRLLTVGNPYFRV** | **60** | **78** | **19** | **HLA-DRB1*09:01** | **0.4** | **A** |
| 49 | GIIIFLKNVNVF | 10 | 21 | 12 | HLA-DRB1*15:01 | 0.397 | NA |
| 50 | GHGIIIFLKNVNVFP | 8 | 22 | 15 | HLA-DRB1*15:01 | 0.3819 | NA |
| 51 | YGHGIIIFLKNVNVFP | 7 | 22 | 16 | HLA-DRB1*15:01 | 0.3738 | NA |
| 52 | NIIYGHGIIIFLKNVNV | 4 | 20 | 17 | HLA-DRB1*15:01 | 0.3725 | NA |
| 53 | IIYGHGIIIFLKNVNVFPI | 5 | 23 | 19 | HLA-DRB1*15:01 | 0.3705 | NA |
| 54 | FYHAGSSRLLTVG | 60 | 72 | 13 | HLA-DRB1*09:01 | 0.3667 | NA |
| 55 | FYHAGSSRLLTVGN | 60 | 73 | 14 | HLA-DRB1*09:01 | 0.3486 | NA |
| 56 | FYHAGSSRLLTVGNPYF | 60 | 76 | 17 | HLA-DRB1*09:01 | 0.3305 | NA |
| 57 | SRTSIFYHAGSSRLLTVG | 55 | 72 | 18 | HLA-DRB1*09:01 | 0.2913 | NA |
| 58 | MSYIHSMNSSIL | 418 | 429 | 12 | HLA-DRB1*09:01 | 0.2875 | NA |
|  |  | 418 | 429 |  | HLA-DRB1*04:05 | 0.2875 | NA |
| 59 | IIYGHGIIIFLKNVNV | 5 | 20 | 16 | HLA-DRB1*15:01 | 0.2838 | NA |
| 60 | VSRTSIFYHAGSSRLLTVG | 54 | 72 | 19 | HLA-DRB1*09:01 | 0.2836 | NA |
| 61 | SRTSIFYHAGSSRLLTVGN | 55 | 73 | 19 | HLA-DRB1*09:01 | 0.2806 | NA |
| 62 | IFYHAGSSRLLTVGNPYFR | 59 | 77 | 19 | HLA-DRB1*09:01 | 0.2663 | NA |
| 63 | HGIIIFLKNVNVF | 9 | 21 | 13 | HLA-DRB1*15:01 | 0.2526 | NA |
| 64 | IYGHGIIIFLKNVNVFPI | 6 | 23 | 18 | HLA-DRB1*15:01 | 0.2513 | NA |
| 65 | MSYIHSMNSSILENWN | 418 | 433 | 16 | HLA-DRB1*04:05 | 0.2478 | NA |
|  |  | 418 | 433 |  | HLA-DRB1*09:01 | 0.2478 | NA |
| 66 | RTSIFYHAGSSRLLTVG | 56 | 72 | 17 | HLA-DRB1*09:01 | 0.2468 | NA |
| 67 | IIYGHGIIIFLKNVNVFP | 5 | 22 | 18 | HLA-DRB1*15:01 | 0.24 | NA |
| 68 | RTSIFYHAGSSRLLTVGN | 56 | 73 | 18 | HLA-DRB1*09:01 | 0.2386 | NA |
| 69 | FYHAGSSRLLTVGNP | 60 | 74 | 15 | HLA-DRB1*09:01 | 0.2374 | NA |
| 70 | MSYIHSMNSSILE | 418 | 430 | 13 | HLA-DRB1*04:05 | 0.2305 | NA |
|  |  | 418 | 430 |  | HLA-DRB1*09:01 | 0.2305 | NA |
|  |  | 418 | 430 |  | HLA-DRB1*13:02 | 0.2305 | NA |
| 71 | FYHAGSSRLLTVGNPY | 60 | 75 | 16 | HLA-DRB1*09:01 | 0.2135 | NA |
| 72 | LTAEVMSYIHSMNSSIL | 413 | 429 | 17 | HLA-DRB1*09:01 | 0.2111 | NA |
| 73 | IYGHGIIIFLKNVNVFPIF | 6 | 24 | 19 | HLA-DRB1*15:01 | 0.1922 | NA |
| 74 | TSIFYHAGSSRLLTVG | 57 | 72 | 16 | HLA-DRB1*09:01 | 0.189 | NA |
| 75 | TSIFYHAGSSRLLTVGN | 57 | 73 | 17 | HLA-DRB1*09:01 | 0.1847 | NA |
| 76 | IFYHAGSSRLLTVGNPYF | 59 | 76 | 18 | HLA-DRB1*09:01 | 0.1732 | NA |
| 77 | TAEVMSYIHSMNSSIL | 414 | 429 | 16 | HLA-DRB1*09:01 | 0.1701 | NA |
|  |  | 414 | 429 |  | HLA-DRB1*04:05 | 0.1701 | NA |
| 78 | SIFYHAGSSRLLTVGNPYF | 58 | 76 | 19 | HLA-DRB1*09:01 | 0.1695 | NA |
| 79 | RTSIFYHAGSSRLLTVGNP | 56 | 74 | 19 | HLA-DRB1*09:01 | 0.165 | NA |
| 80 | MSYIHSMNSSILEN | 418 | 431 | 14 | HLA-DRB1*04:05 | 0.1541 | NA |
|  |  | 418 | 431 |  | HLA-DRB1*09:01 | 0.1541 | NA |
|  |  | 418 | 431 |  | HLA-DRB1*13:02 | 0.1541 | NA |
| 81 | IFYHAGSSRLLTVGN | 59 | 73 | 15 | HLA-DRB1*09:01 | 0.1499 | NA |
| 82 | IFYHAGSSRLLTVG | 59 | 72 | 14 | HLA-DRB1*09:01 | 0.1498 | NA |
| 83 | SIFYHAGSSRLLTVG | 58 | 72 | 15 | HLA-DRB1*09:01 | 0.1451 | NA |
| 84 | SIFYHAGSSRLLTVGN | 58 | 73 | 16 | HLA-DRB1*09:01 | 0.145 | NA |
| 85 | TAEVMSYIHSMNSSILE | 414 | 430 | 17 | HLA-DRB1*09:01 | 0.1381 | NA |
|  |  | 414 | 430 |  | HLA-DRB1*04:05 | 0.1381 | NA |
| 86 | IYGHGIIIFLKNVNV | 6 | 20 | 15 | HLA-DRB1*15:01 | 0.1271 | NA |
| 87 | YGHGIIIFLKNVNVF | 7 | 21 | 15 | HLA-DRB1*15:01 | 0.1228 | NA |
| 88 | AEVMSYIHSMNSSI | 415 | 428 | 14 | HLA-DRB1*04:05 | 0.12227 | NA |
|  |  | 415 | 429 |  | HLA-DRB1*09:01 | 0.12227 | NA |
|  |  | 415 | 429 |  | HLA-DRB1*04:05 | 0.12227 | NA |
|  |  | 415 | 429 |  | HLA-DRB1*13:02 | 0.12227 | NA |
| 89 | SYIHSMNSSILENWN | 419 | 433 | 15 | HLA-DRB1*09:01 | 0.1167 | NA |
| 90 | NIIYGHGIIIFLKNVNVF | 4 | 21 | 18 | HLA-DRB1*15:01 | 0.1145 | NA |
| 91 | VMSYIHSMNSSILENWN | 417 | 433 | 17 | HLA-DRB1*04:05 | 0.1138 | NA |
| 92 | GHGIIIFLKNVNVF | 8 | 21 | 14 | HLA-DRB1*15:01 | 0.1091 | NA |
| 93 | TSIFYHAGSSRLLTVGNP | 57 | 74 | 18 | HLA-DRB1*09:01 | 0.109 | NA |
| 94 | IYGHGIIIFLKNVNVFP | 6 | 22 | 17 | HLA-DRB1*15:01 | 0.1038 | NA |
| 95 | VMSYIHSMNSSIL | 417 | 429 | 13 | HLA-DRB1*09:01 | 0.09765 | NA |
|  |  | 417 | 429 |  | HLA-DRB1*04:05 | 0.09765 | NA |
|  |  | 417 | 429 |  | HLA-DRB1*13:02 | 0.09765 | NA |
| 96 | TSIFYHAGSSRLLTVGNPY | 57 | 75 | 19 | HLA-DRB1*09:01 | 0.0974 | NA |
| 97 | EVMSYIHSMNSSI | 416 | 428 | 13 | HLA-DRB1*04:05 | 0.0889 | NA |
| 98 | AEVMSYIHSMNSSILE | 415 | 430 | 16 | HLA-DRB1*09:01 | 0.0779 | NA |
|  |  | 415 | 430 |  | HLA-DRB1*04:05 | 0.0779 | NA |
| 99 | EVMSYIHSMNSSIL | 416 | 429 | 14 | HLA-DRB1*09:01 | 0.0777 | NA |
|  |  | 416 | 429 |  | HLA-DRB1*04:05 | 0.0777 | NA |
|  |  | 416 | 429 |  | HLA-DRB1*13:02 | 0.0777 | NA |
| 100 | SIFYHAGSSRLLTVGNP | 58 | 74 | 17 | HLA-DRB1*09:01 | 0.0662 | NA |
| 101 | IFYHAGSSRLLTVGNP | 59 | 74 | 16 | HLA-DRB1*09:01 | 0.0633 | NA |
| 102 | VMSYIHSMNSSILE | 417 | 430 | 14 | HLA-DRB1*09:01 | 0.0627 | NA |
|  |  | 417 | 430 |  | HLA-DRB1*04:05 | 0.0627 | NA |
|  |  | 417 | 430 |  | HLA-DRB1*13:02 | 0.0627 | NA |
| 103 | SYIHSMNSSILE | 419 | 430 | 12 | HLA-DRB1*09:01 | 0.057 | NA |
| 104 | SIFYHAGSSRLLTVGNPY | 58 | 75 | 18 | HLA-DRB1*09:01 | 0.0569 | NA |
| 105 | MSYIHSMNSSILENW | 418 | 432 | 15 | HLA-DRB1*04:05 | 0.0568 | NA |
|  |  | 418 | 432 |  | HLA-DRB1*09:01 | 0.0568 | NA |
|  |  | 418 | 432 |  | HLA-DRB1*13:02 | 0.0568 | NA |
| 106 | IFYHAGSSRLLTVGNPY | 59 | 75 | 17 | HLA-DRB1*09:01 | 0.0533 | NA |
| 107 | EVMSYIHSMNSSILE | 416 | 430 | 15 | HLA-DRB1*04:05 | 0.0476 | NA |
|  |  | 416 | 430 |  | HLA-DRB1*09:01 | 0.0476 | NA |
|  |  | 416 | 430 |  | HLA-DRB1*13:02 | 0.0476 | NA |
| 108 | YVSRTSIFYHAGSSRLLTV | 53 | 71 | 19 | HLA-DRB1*09:01 | 0.0455 | NA |
| 109 | AEVMSYIHSMNSSILEN | 415 | 431 | 17 | HLA-DRB1*09:01 | 0.0262 | NA |
|  |  | 415 | 431 |  | HLA-DRB1*04:05 | 0.0262 | NA |
| 110 | HNIIYGHGIIIFLKNVNVF | 3 | 21 | 19 | HLA-DRB1*15:01 | 0.0058 | NA |
| 111 | VMSYIHSMNSSILEN | 417 | 431 | 15 | HLA-DRB1*04:05 | 0.0052 | NA |
|  |  | 417 | 431 |  | HLA-DRB1*09:01 | 0.0052 | NA |
|  |  | 417 | 431 |  | HLA-DRB1*13:02 | 0.0052 | NA |
| 112 | IIYGHGIIIFLKNVNVF | 5 | 21 | 17 | HLA-DRB1*15:01 | 0.00153 | NA |
| 113 | DYVSRTSIFYHAGSSRLL | 52 | 69 | 18 | HLA-DRB1*09:01 | -0.0038 | NA |
| 114 | EVMSYIHSMNSSILEN | 416 | 431 | 16 | HLA-DRB1*04:05 | -0.0052 | NA |
|  |  | 416 | 431 |  | HLA-DRB1*09:01 | -0.0052 | NA |
|  |  | 416 | 431 |  | HLA-DRB1*13:02 | -0.0052 | NA |
| 115 | VSRTSIFYHAGSSRLLTV | 54 | 71 | 18 | HLA-DRB1*09:01 | -0.0057 | NA |
| 116 | SYIHSMNSSILEN | 419 | 431 | 13 | HLA-DRB1*09:01 | -0.0092 | NA |
|  |  | 419 | 431 |  | HLA-DRB1*04:05 | -0.0092 | NA |
| 117 | SRTSIFYHAGSSRLLTV | 55 | 71 | 17 | HLA-DRB1*09:01 | -0.018 | NA |
| 118 | YVSRTSIFYHAGSSRLL | 53 | 69 | 17 | HLA-DRB1*09:01 | -0.0388 | NA |
| 119 | DYVSRTSIFYHAGSSRLLT | 52 | 70 | 19 | HLA-DRB1*09:01 | -0.058 | NA |
| 120 | DDYVSRTSIFYHAGSSRLL | 51 | 69 | 19 | HLA-DRB1*09:01 | -0.0665 | NA |
| 121 | VMSYIHSMNSSILENW | 417 | 432 | 16 | HLA-DRB1*04:05 | -0.0728 | NA |
|  |  | 417 | 432 |  | HLA-DRB1*09:01 | -0.0728 | NA |
|  |  | 417 | 432 |  | HLA-DRB1*13:02 | -0.0728 | NA |
| 122 | EVMSYIHSMNSSILENW | 416 | 432 | 17 | HLA-DRB1*04:05 | -0.0764 | NA |
| 123 | RTSIFYHAGSSRLLTV | 56 | 71 | 16 | HLA-DRB1*09:01 | -0.0896 | NA |
| 124 | YVSRTSIFYHAGSSRLLT | 53 | 70 | 18 | HLA-DRB1*09:01 | -0.0951 | NA |
| 125 | SYIHSMNSSILENW | 419 | 432 | 14 | HLA-DRB1*09:01 | -0.1023 | NA |
|  |  | 419 | 432 |  | HLA-DRB1*04:05 | -0.1023 | NA |
| 126 | VSRTSIFYHAGSSRLL | 54 | 69 | 16 | HLA-DRB1*09:01 | -0.1046 | NA |
| 127 | FYHAGSSRLLTV | 60 | 71 | 12 | HLA-DRB1*09:01 | -0.1086 | NA |
| 128 | SRTSIFYHAGSSRLL | 55 | 69 | 15 | HLA-DRB1*09:01 | -0.1267 | NA |
| 129 | IYGHGIIIFLKNVNVF | 6 | 21 | 16 | HLA-DRB1*15:01 | -0.1483 | NA |
| 130 | VSRTSIFYHAGSSRLLT | 54 | 70 | 17 | HLA-DRB1*09:01 | -0.16 | NA |
| 131 | TSIFYHAGSSRLLTV | 57 | 71 | 15 | HLA-DRB1*09:01 | -0.18 | NA |
| 132 | SRTSIFYHAGSSRLLT | 55 | 70 | 16 | HLA-DRB1*09:01 | -0.1848 | NA |
| 133 | RTSIFYHAGSSRLL | 56 | 69 | 14 | HLA-DRB1*09:01 | -0.2201 | NA |
| 134 | SIFYHAGSSRLLTV | 58 | 71 | 14 | HLA-DRB1*09:01 | -0.2624 | NA |
| 135 | RTSIFYHAGSSRLLT | 56 | 70 | 15 | HLA-DRB1*09:01 | -0.2758 | NA |
| 136 | IFYHAGSSRLLTV | 59 | 71 | 13 | HLA-DRB1*09:01 | -0.298 | NA |
| 137 | TSIFYHAGSSRLL | 57 | 69 | 13 | HLA-DRB1*09:01 | -0.3401 | NA |
| 138 | TSIFYHAGSSRLLT | 57 | 70 | 14 | HLA-DRB1*09:01 | -0.3906 | NA |
| 139 | SIFYHAGSSRLL | 58 | 69 | 12 | HLA-DRB1*09:01 | -0.4605 | NA |
| 140 | SIFYHAGSSRLLT | 58 | 70 | 13 | HLA-DRB1*09:01 | -0.503 | NA |
| 141 | IFYHAGSSRLLT | 59 | 70 | 12 | HLA-DRB1*09:01 | -0.5684 | NA |

Supplementary Table 4 VaxiJen analysis of peptides with IEDB HLA-1 binding scores (to HPV-related high frequency HLA alleles in West Java) ≥ 0.6. Peptides with VaxiJen scores ≥1 were analysed further. Those in bold are antigenic peptides.

| **No** | **Peptide** | **Start** | **End** | **Length** | **Allele** | **VaxiJen Score** | **Antigenic (A)/Non-Antigenic (NA)** |
| --- | --- | --- | --- | --- | --- | --- | --- |
| 1 | **VDYKQTQL** | **176** | **183** | **8** | **HLA-B*37:01** | **2.0021** | **A** |
| 2 | **EEYDLQFIF** | **398** | **406** | **9** | **HLA-B*40:02** | **1.7384** | **A** |
|  |  |  |  |  | **HLA-B*40:01** |  |  |
| 3 | **YDLQFIFQL** | **400** | **408** | **9** | **HLA-B*40:02** | **1.6395** | **A** |
|  |  |  |  |  | **HLA-B*37:01** |  |  |
| 4 | **TTRSTNLTL** | **365** | **373** | **9** | **HLA-B*15:17** | **1.5882** | **A** |
| 5 | **WTVDLKEKF** | **477** | **485** | **9** | **HLA-B*15:17** | **1.2928** | **A** |
| 6 | **LTVGNPYFR** | **69** | **77** | **9** | **HLA-A*33:03** | **1.2652** | **A** |
| 7 | **VDTTRSTNL** | **363** | **371** | **9** | **HLA-B*37:01** | **1.247** | **A** |
| 8 | **KLKFWTVDLK** | **473** | **482** | **10** | **HLA-A*03:01** | **1.1434** | **A** |
| 9 | **MEIGRGQPL** | **132** | **140** | **9** | **HLA-B*40:01** | **0.9861** | **A** |
|  |  |  |  |  | **HLA-B*40:02** |  |  |
| 10 | **TPPEKQDPY** | **463** | **471** | **9** | **HLA-B*35:01** | **0.9829** | **A** |
| 11 | **RPAKRVRI** | **525** | **532** | **8** | **HLA-B*07:05** | **0.933** | **A** |
|  |  |  |  |  | **HLA-B*07:02** |  |  |
| 12 | **TQNPVPGTY** | **377** | **385** | **9** | **HLA-B*15:01** | **0.8973** | **A** |
|  |  |  |  |  | **HLA-B*15:25** |  |  |
|  |  |  |  |  | **HLA-B*15:02** |  |  |
|  |  |  |  |  | **HLA-B*15:12** |  |  |
|  |  |  |  |  | **HLA-B*15:32** |  |  |
|  |  |  |  |  | **HLA-B*15:21** |  |  |
|  |  |  |  |  | **HLA-B*15:13** |  |  |
| 13 | **KFWTVDLKEKF** | **475** | **485** | **11** | **HLA-B*15:17** | **0.8763** | **A** |
| 14 | **ITLTAEVMSY** | **411** | **420** | **10** | **HLA-B*15:17** | **0.8453** | **A** |
| 15 | **RVVPSGAGNK** | **77** | **86** | **10** | **HLA-A*03:01** | **0.8424** | **A** |
| 16 | **NVFPIFLQM** | **19** | **27** | **9** | **HLA-B*15:17** | **0.8028** | **A** |
|  |  |  |  |  | **HLA-B*15:02** |  |  |
| 17 | **STQNPVPGTY** | **376** | **385** | **10** | **HLA-B*15:01** | **0.778** | **A** |
|  |  |  |  |  | **HLA-B*15:17** |  |  |
|  |  |  |  |  | **HLA-B*15:25** |  |  |
|  |  |  |  |  | **HLA-B*15:12** |  |  |
|  |  |  |  |  | **HLA-B*15:32** |  |  |
|  |  |  |  |  | **HLA-B*15:02** |  |  |
| 18 | **FPIFLQMAL** | **21** | **29** | **9** | **HLA-B*35:01** | **0.7776** | **A** |
|  |  |  |  |  | **HLA-B*07:05** |  |  |
|  |  |  |  |  | **HLA-B*07:02** |  |  |
| 19 | **SSILENWNF** | **426** | **434** | **9** | **HLA-B*15:17** | **0.765** | **A** |
|  |  |  |  |  | **HLA-B*58:01** |  |  |
| 20 | **QPGDCPPLEL** | **208** | **217** | **10** | **HLA-B*07:05** | **0.7224** | **A** |
| 21 | **VPPPPTTSL** | **436** | **444** | **9** | **HLA-B*07:05** | **0.7072** | **A** |
|  |  |  |  |  | **HLA-B*07:02** |  |  |
|  |  |  |  |  | **HLA-B*35:01** |  |  |
| 22 | **ASTQNPVPGTY** | **375** | **385** | **11** | **HLA-B*15:01** | **0.7019** | **A** |
|  |  |  |  |  | **HLA-B*15:25** |  |  |
|  |  |  |  |  | **HLA-B*15:17** |  |  |
|  |  |  |  |  | **HLA-B*15:12** |  |  |
| 23 | **YLQMSADPY** | **261** | **269** | **9** | **HLA-B*15:02** | **0.6908** | **A** |
| 24 | **YIKGTSANM** | **303** | **311** | **9** | **HLA-B*15:02** | **0.6799** | **A** |
| 25 | **NMRETPGSCVY** | **310** | **320** | **11** | **HLA-B*15:02** | **0.6681** | **A** |
| 26 | **GTYDPTKFKHY** | **383** | **393** | **11** | **HLA-B*15:17** | **0.6629** | **A** |
| 27 | **VPPPPTTSLV** | **436** | **445** | **10** | **HLA-B*07:05** | **0.6486** | **A** |
| 28 | **SPSPSGSIT** | **321** | **329** | **9** | **HLA-B*07:05** | **0.6293** | **A** |
| 29 | **GVPPPPTTSL** | **435** | **444** | **10** | **HLA-B*07:05** | **0.6223** | **A** |
|  |  |  |  |  | **HLA-B*07:02** |  |  |
| 30 | **KAQGHNNGICW** | **344** | **354** | **11** | **HLA-B*58:01** | **0.5989** | **A** |
| 31 | **FGVPPPPTTSL** | **434** | **444** | **11** | **HLA-B*07:05** | **0.5527** | **A** |
|  |  |  |  |  | **HLA-B*07:02** |  |  |
| 32 | **GLSGHPFYNK** | **143** | **152** | **10** | **HLA-A*03:01** | **0.5524** | **A** |
| 33 | **STASRPAKR** | **521** | **529** | **9** | **HLA-A*33:03** | **0.5483** | **A** |
| 34 | **RLLTVGNPY** | **67** | **75** | **9** | **HLA-B*15:25** | **0.5385** | **A** |
|  |  |  |  |  | **HLA-B*15:01** |  |  |
| 35 | **GTYDPTKFK** | **383** | **391** | **9** | **HLA-A*03:01** | **0.4814** | **A** |
| 36 | **VPGTYDPTKFK** | **381** | **391** | **11** | **HLA-A*03:01** | **0.4638** | **A** |
| 37 | **SPSGSITTSDSQL** | **323** | **335** | **13** | **HLA-B*07:05** | **0.4126** | **A** |
| 38 | **YLPPPSVARV** | **38** | **47** | **10** | **HLA-A*02:03** | **0.4104** | **A** |
|  |  |  |  |  | **HLA-A*02:01** |  |  |
|  |  |  |  |  | **HLA-A*02:06** |  |  |
| 39 | **TTSLVDTYR** | **441** | **449** | **9** | **HLA-A*33:03** | **0.4094** | **A** |
| 40 | **ALPDPNKFGL** | **103** | **112** | **10** | **HLA-B*07:05** | **0.4074** | **A** |
| 41 | **LTAEVMSY** | **413** | **420** | **8** | **HLA-B*15:17** | **0.4038** | **A** |
| 42 | YVSRTSIFY | 53 | 61 | 9 | HLA-B*15:17 | 0.3935 | NA |
|  |  |  |  |  | HLA-B*15:02 |  |  |
|  |  |  |  |  | HLA-B*15:25 |  |  |
|  |  |  |  |  | HLA-B*35:01 |  |  |
|  |  |  |  |  | HLA-B*15:01 |  |  |
| 43 | TLTAEVMSY | 412 | 420 | 9 | HLA-B*15:02 | 0.372 | NA |
|  |  |  |  |  | HLA-B*15:25 |  |  |
|  |  |  |  |  | HLA-B*15:01 |  |  |
|  |  |  |  |  | HLA-B*15:21 |  |  |
| 44 | LPDPNKFGL | 104 | 112 | 9 | HLA-B*07:05 | 0.3282 | NA |
|  |  |  |  |  | HLA-B*07:02 |  |  |
|  |  |  |  |  | HLA-B*35:01 |  |  |
| 45 | QAVPKVSAY | 87 | 95 | 9 | HLA-B*35:01 | 0.3232 | NA |
|  |  |  |  |  | HLA-B*15:02 |  |  |
|  |  |  |  |  | HLA-B*15:17 |  |  |
|  |  |  |  |  | HLA-B*15:25 |  |  |
|  |  |  |  |  | HLA-B*15:01 |  |  |
|  |  |  |  |  | HLA-B*15:21 |  |  |
|  |  |  |  |  | HLA-B*15:12 |  |  |
|  |  |  |  |  | HLA-B*15:32 |  |  |
|  |  |  |  |  | HLA-B*15:13 |  |  |
| 46 | YLPPPSVAR | 38 | 46 | 9 | HLA-A*33:03 | 0.3103 | NA |
| 47 | GEHWAKGTL | 193 | 201 | 9 | HLA-B*40:01 | 0.3085 | NA |
|  |  |  |  |  | HLA-B*40:02 |  |  |
| 48 | SPSPSGSI | 321 | 328 | 8 | HLA-B*07:05 | 0.2827 | NA |
|  |  |  |  |  | HLA-B*07:02 |  |  |
| 49 | VSAYQYRVF | 92 | 100 | 9 | HLA-B*15:17 | 0.2689 | NA |
| 50 | GTYDPTKF | 383 | 390 | 8 | HLA-B*15:17 | 0.2638 | NA |
| 51 | LTAEVMSYI | 413 | 421 | 9 | HLA-B*15:17 | 0.2565 | NA |
| 52 | GSITTSDSQLF | 326 | 336 | 11 | HLA-B*15:17 | 0.2338 | NA |
| 53 | QLFNKPYWLHK | 334 | 344 | 11 | HLA-A*03:01 | 0.2168 | NA |
| 54 | FGLPDSTIY | 110 | 118 | 9 | HLA-B*35:01 | 0.1881 | NA |
| 55 | IHSMNSSIL | 421 | 429 | 9 | HLA-B*15:10 | 0.1857 | NA |
| 56 | QLFNKPYWL | 334 | 342 | 9 | HLA-A*02:01 | 0.1842 | NA |
| 57 | QAVPKVSAYQY | 87 | 97 | 11 | HLA-B*15:17 | 0.1712 | NA |
| 58 | TVYLPPPSV | 36 | 44 | 9 | HLA-A*02:06 | 0.1695 | NA |
|  |  |  |  |  | HLA-A*02:03 |  |  |
|  |  |  |  |  | HLA-A*02:01 |  |  |
| 59 | RPAASTSTA | 515 | 523 | 9 | HLA-B*07:05 | 0.1505 | NA |
|  |  |  |  |  | HLA-B*07:02 |  |  |
| 60 | RPSDSTVYL | 31 | 39 | 9 | HLA-B*07:05 | 0.1266 | NA |
|  |  |  |  |  | HLA-B*07:02 |  |  |
|  |  |  |  |  | HLA-B*35:01 |  |  |
| 61 | TVYLPPPSVAR | 36 | 46 | 11 | HLA-A*33:03 | 0.1154 | NA |
| 62 | VYLPPPSVAR | 37 | 46 | 10 | HLA-A*33:03 | 0.1023 | NA |
| 63 | TTSDSQLFNKPYW | 329 | 341 | 13 | HLA-B*58:01 | 0.0839 | NA |
| 64 | RVALPDPNK | 101 | 109 | 9 | HLA-A*03:01 | 0.0447 | NA |
| 65 | ITTSDSQLF | 328 | 336 | 9 | HLA-B*15:17 | 0.0385 | NA |
|  |  |  |  |  | HLA-B*58:01 |  |  |
| 66 | TSLVDTYRF | 442 | 450 | 9 | HLA-B*15:17 | 0.0321 | NA |
|  |  |  |  |  | HLA-B*58:01 |  |  |
| 67 | SLVDTYRFV | 443 | 451 | 9 | HLA-A*02:03 | 0.0137 | NA |
|  |  |  |  |  | HLA-A*02:01 |  |  |
|  |  |  |  |  | HLA-A*02:06 |  |  |
| 68 | AEVMSYIHSM | 415 | 424 | 10 | HLA-B*40:02 | -0.0021 | NA |
|  |  |  |  |  | HLA-B*40:01 |  |  |
| 69 | IIYGHGIIIFLK | 5 | 16 | 12 | HLA-A*03:01 | -0.0072 | NA |
| 70 | REQLFARHF | 279 | 287 | 9 | HLA-B*40:02 | -0.0092 | NA |
| 71 | MSADPYGDSMF | 264 | 274 | 11 | HLA-B*15:17 | -0.0128 | NA |
| 72 | YHAGSSRL | 61 | 68 | 8 | HLA-B*15:10 | -0.0248 | NA |
| 73 | VPKVSAYQY | 89 | 97 | 9 | HLA-B*35:01 | -0.0555 | NA |
|  |  |  |  |  | HLA-B*15:02 |  |  |
| 74 | EVMSYIHSM | 416 | 424 | 9 | HLA-B*15:02 | -0.0795 | NA |
|  |  |  |  |  | HLA-B*35:01 |  |  |
| 75 | MSADPYGDSMFF | 264 | 275 | 12 | HLA-B*15:17 | -0.082 | NA |
| 76 | SADPYGDSMFF | 265 | 275 | 11 | HLA-B*15:17 | -0.1431 | NA |
| 77 | HSMNSSILENW | 422 | 432 | 11 | HLA-B*58:01 | -0.1575 | NA |
|  |  |  |  |  | HLA-B*15:17 |  |  |
| 78 | KQAVPKVSAYQY | 86 | 97 | 12 | HLA-B*15:01 | -0.1933 | NA |
| 79 | RVALPDPNKF | 101 | 110 | 10 | HLA-B*15:17 | -0.2093 | NA |
|  |  |  |  |  | HLA-B*58:01 |  |  |
| 80 | KQAVPKVSAY | 86 | 95 | 10 | HLA-B*15:01 | -0.2162 | NA |
|  |  |  |  |  | HLA-B*15:25 |  |  |
|  |  |  |  |  | HLA-B*15:32 |  |  |
|  |  |  |  |  | HLA-B*15:12 |  |  |
|  |  |  |  |  | HLA-B*15:17 |  |  |
| 81 | IFYHAGSSR | 59 | 67 | 9 | HLA-A*33:03 | -0.239 | NA |
| 82 | DPYGDSMFF | 267 | 275 | 9 | HLA-B*35:01 | -0.2414 | NA |
| 83 | VALPDPNKF | 102 | 110 | 9 | HLA-B*15:17 | -0.2866 | NA |
|  |  |  |  |  | HLA-B*58:01 |  |  |
|  |  |  |  |  | HLA-B*35:01 |  |  |
| 84 | MNSSILENW | 424 | 432 | 9 | HLA-B*58:01 | -0.2954 | NA |
| 85 | ALWRPSDSTVYL | 28 | 39 | 12 | HLA-A*02:01 | -0.3178 | NA |
| 86 | TIYNPETQR | 116 | 124 | 9 | HLA-A*33:03 | -0.3554 | NA |
|  |  |  |  |  | HLA-A*03:01 |  |  |
| 87 | YHAGSSRLL | 61 | 69 | 9 | HLA-B*15:10 | -0.3745 | NA |
| 88 | ALWRPSDSTVY | 28 | 38 | 11 | HLA-B*15:25 | -0.417 | NA |
|  |  |  |  |  | HLA-B*15:01 |  |  |
| 89 | LDQYPLGRKF | 489 | 498 | 10 | HLA-B*15:01 | -0.4185 | NA |
|  |  |  |  |  | HLA-B*15:25 |  |  |
| 90 | DQYPLGRKF | 490 | 498 | 9 | HLA-B*15:02 | -0.4721 | NA |
|  |  |  |  |  | HLA-B*15:13 |  |  |
|  |  |  |  |  | HLA-B*15:12 |  |  |
| 91 | RVFRVALPDPNK | 98 | 109 | 12 | HLA-A*03:01 | -0.4974 | NA |
| 92 | GCVPAIGEHW | 187 | 196 | 10 | HLA-B*58:01 | -0.506 | NA |
| 93 | SAYQYRVFR | 93 | 101 | 9 | HLA-A*33:03 | -0.5358 | NA |
| 94 | YPLGRKFL | 492 | 499 | 8 | HLA-B*07:05 | -0.5648 | NA |
| 95 | LFARHFWNR | 282 | 290 | 9 | HLA-A*33:03 | -1.1942 | NA |

Supplementary Table 5 VaxiJen analysis of peptides with IEDB HLA-2 adjusted percentile rank (upon binding to HPV-related high frequency HLA alleles in West Java) < 1. Peptides with VaxiJen scores ≥1 were analysed further. Those in bold are antigenic peptides.

| **No** | **Peptide** | **start** | **end** | **length** | **Alleles** | **VaxiJen Score** | **Antigenic/ Non-Antigenic** |
| --- | --- | --- | --- | --- | --- | --- | --- |
| 1 | **GLRRRPTIGPRKR** | **503** | **515** | **13** | **HLA-DRB1*04:02** | **2.0738** | **A** |
| 2 | **AGLRRRPTIGPRKR** | **502** | **515** | **14** | **HLA-DRB1*04:02** | **2.0411** | **A** |
| 3 | **LRRRPTIGPRKRP** | **504** | **516** | **13** | **HLA-DRB1*04:02** | **1.9788** | **A** |
| 4 | **VQAGLRRRPTIGPRKR** | **500** | **515** | **16** | **HLA-DRB1*04:02** | **1.8995** | **A** |
| 5 | **GLRRRPTIGPRKRP** | **503** | **516** | **14** | **HLA-DRB1*04:02** | **1.8611** | **A** |
| 6 | **AGLRRRPTIGPRKRP** | **502** | **516** | **15** | **HLA-DRB1*04:02** | **1.8494** | **A** |
| 7 | **VQAGLRRRPTIGPR** | **500** | **513** | **14** | **HLA-DRB1*04:02** | **1.8122** | **A** |
| 8 | **QAGLRRRPTIGPRKR** | **501** | **515** | **15** | **HLA-DRB1*04:02** | **1.8078** | **A** |
| 9 | **LRRRPTIGPRKRPA** | **504** | **517** | **14** | **HLA-DRB1*04:02** | **1.7785** | **A** |
| 10 | **AGLRRRPTIGPRK** | **502** | **514** | **13** | **HLA-DRB1*04:02** | **1.7631** | **A** |
| 11 | **AGLRRRPTIGPRKRPA** | **502** | **517** | **16** | **HLA-DRB1*04:02** | **1.6938** | **A** |
| 12 | **QAGLRRRPTIGPR** | **501** | **513** | **13** | **HLA-DRB1*04:02** | **1.6929** | **A** |
| 13 | **GLRRRPTIGPRKRPA** | **503** | **517** | **15** | **HLA-DRB1*04:02** | **1.6909** | **A** |
| 14 | **LRRRPTIGPRKRPAA** | **504** | **518** | **15** | **HLA-DRB1*04:02** | **1.6606** | **A** |
| 15 | **VQAGLRRRPTIGPRK** | **500** | **514** | **15** | **HLA-DRB1*04:02** | **1.6539** | **A** |
| 16 | **QAGLRRRPTIGPRKRP** | **501** | **516** | **16** | **HLA-DRB1*04:02** | **1.6522** | **A** |
| 17 | **GLRRRPTIGPRKRPAA** | **503** | **518** | **16** | **HLA-DRB1*04:02** | **1.5902** | **A** |
| 18 | **VQAGLRRRPTIGP** | **500** | **512** | **13** | **HLA-DRB1*04:02** | **1.5776** | **A** |
| 19 | **LVQAGLRRRPTIGPR** | **499** | **513** | **15** | **HLA-DRB1*04:02** | **1.5675** | **A** |
| 20 | **QAGLRRRPTIGPRK** | **501** | **514** | **14** | **HLA-DRB1*04:02** | **1.5305** | **A** |
| 21 | **LRRRPTIGPRKRPAAS** | **504** | **519** | **16** | **HLA-DRB1*04:02** | **1.5179** | **A** |
| 22 | **FLVQAGLRRRPTIGPR** | **498** | **513** | **16** | **HLA-DRB1*04:02** | **1.4645** | **A** |
| 23 | **LVQAGLRRRPTIGPRK** | **499** | **514** | **16** | **HLA-DRB1*04:02** | **1.441** | **A** |
| 24 | **LVQAGLRRRPTIGP** | **499** | **512** | **14** | **HLA-DRB1*04:02** | **1.3345** | **A** |
| 25 | **FLVQAGLRRRPTIGP** | **498** | **512** | **15** | **HLA-DRB1*04:02** | **1.244** | **A** |
| 26 | **KFLVQAGLRRRPTIGP** | **497** | **512** | **16** | **HLA-DRB1*04:02** | **0.9855** | **A** |
| 27 | **GNPYFRVVPSGAG** | **72** | **84** | **13** | **HLA-DRB1*09:01** | **0.9729** | **A** |
| 28 | **TVGNPYFRVVPSGAG** | **70** | **84** | **15** | **HLA-DRB1*09:01** | **0.9302** | **A** |
| 29 | **VGNPYFRVVPSGAG** | **71** | **84** | **14** | **HLA-DRB1*09:01** | **0.9195** | **A** |
| 30 | **GNPYFRVVPSGAGNK** | **72** | **86** | **15** | **HLA-DRB1*09:01** | **0.8915** | **A** |
| 31 | **NPYFRVVPSGAGNK** | **73** | **86** | **14** | **HLA-DRB1*09:01** | **0.8789** | **A** |
| 32 | **IIIFLKNVNVFPI** | **11** | **23** | **13** | **HLA-DRB1*13:02** | **0.8701** | **A** |
|  |  |  |  |  | **HLA-DRB1*15:01** |  |  |
| 33 | **GNPYFRVVPSGAGN** | **72** | **85** | **14** | **HLA-DRB1*09:01** | **0.8452** | **A** |
| 34 | **GIIIFLKNVNVFPI** | **10** | **23** | **14** | **HLA-DRB1*15:01** | **0.8396** | **A** |
|  |  |  |  |  | **HLA-DRB1*13:02** |  |  |
| 35 | **NPYFRVVPSGAGN** | **73** | **85** | **13** | **HLA-DRB1*09:01** | **0.8211** | **A** |
| 36 | **IIFLKNVNVFPIF** | **12** | **24** | **13** | **HLA-DRB1*13:02** | **0.8128** | **A** |
| 37 | **VGNPYFRVVPSGAGN** | **71** | **85** | **15** | **HLA-DRB1*09:01** | **0.8093** | **A** |
| 38 | **IIFLKNVNVFPIFLQ** | **12** | **26** | **15** | **HLA-DRB1*13:02** | **0.7925** | **A** |
| 39 | **IIFLKNVNVFPIFL** | **12** | **25** | **14** | **HLA-DRB1*13:02** | **0.7787** | **A** |
| 40 | **IIIFLKNVNVFPIFLQM** | **11** | **27** | **17** | **HLA-DRB1*15:01** | **0.7552** | **A** |
| 41 | **GIIIFLKNVNVFPIFLQM** | **10** | **27** | **18** | **HLA-DRB1*15:01** | **0.7477** | **A** |
| 42 | **IIIFLKNVNVFPIF** | **11** | **24** | **14** | **HLA-DRB1*13:02** | **0.7318** | **A** |
|  |  |  |  |  | **HLA-DRB1*15:01** |  |  |
| 43 | **IIIFLKNVNVFPIFLQ** | **11** | **26** | **16** | **HLA-DRB1*15:01** | **0.7254** | **A** |
|  |  |  |  |  | **HLA-DRB1*13:02** |  |  |
| 44 | **GIIIFLKNVNVFPIFLQ** | **10** | **26** | **17** | **HLA-DRB1*15:01** | **0.7176** | **A** |
| 45 | **GIIIFLKNVNVFPIF** | **10** | **24** | **15** | **HLA-DRB1*15:01** | **0.7168** | **A** |
|  |  |  |  |  | **HLA-DRB1*13:02** |  |  |
|  |  |  |  |  | **HLA-DRB1*04:03** |  |  |
|  |  |  |  |  | **HLA-DRB1*04:06** |  |  |
| 46 | **IIIFLKNVNVFPIFL** | **11** | **25** | **15** | **HLA-DRB1*13:02** | **0.7079** | **A** |
|  |  |  |  |  | **HLA-DRB1*15:01** |  |  |
| 47 | **GIIIFLKNVNVFPIFL** | **10** | **25** | **16** | **HLA-DRB1*15:01** | **0.6992** | **A** |
|  |  |  |  |  | **HLA-DRB1*13:02** |  |  |
| 48 | **GIIIFLKNVNVFP** | **10** | **22** | **13** | **HLA-DRB1*15:01** | **0.696** | **A** |
| 49 | **HGIIIFLKNVNVFPI** | **9** | **23** | **15** | **HLA-DRB1*15:01** | **0.6794** | **A** |
|  |  |  |  |  | **HLA-DRB1*13:02** |  |  |
| 50 | **HGIIIFLKNVNV** | **9** | **20** | **12** | **HLA-DRB1*15:01** | **0.6544** | **A** |
| 51 | **HGIIIFLKNVNVFPIFLQM** | **9** | **27** | **19** | **HLA-DRB1*15:01** | **0.6289** | **A** |
| 52 | **HGIIIFLKNVNVFPIFLQ** | **9** | **26** | **18** | **HLA-DRB1*15:01** | **0.5935** | **A** |
| 53 | **HGIIIFLKNVNVFPIF** | **9** | **24** | **16** | **HLA-DRB1*15:01** | **0.5756** | **A** |
|  |  |  |  |  | **HLA-DRB1*13:02** |  |  |
| 54 | **MSYIHSMNSSILENWNF** | **418** | **434** | **17** | **HLA-DRB1*04:05** | **0.5693** | **A** |
| 55 | **HGIIIFLKNVNVFPIFL** | **9** | **25** | **17** | **HLA-DRB1*15:01** | **0.5688** | **A** |
| 56 | **HGIIIFLKNVNVFP** | **9** | **22** | **14** | **HLA-DRB1*15:01** | **0.535** | **A** |
| 57 | **GHGIIIFLKNVNVFPI** | **8** | **23** | **16** | **HLA-DRB1*15:01** | **0.5293** | **A** |
|  |  |  |  |  | **HLA-DRB1*13:02** |  |  |
| 58 | **YGHGIIIFLKNVNVFPI** | **7** | **23** | **17** | **HLA-DRB1*15:01** | **0.5122** | **A** |
| 59 | **GHGIIIFLKNVNVFPIFLQ** | **8** | **26** | **19** | **HLA-DRB1*15:01** | **0.4788** | **A** |
| 60 | **SYIHSMNSSILENWNF** | **419** | **434** | **16** | **HLA-DRB1*09:01** | **0.471** | **A** |
| 61 | **GHGIIIFLKNVNV** | **8** | **20** | **13** | **HLA-DRB1*15:01** | **0.4579** | **A** |
| 62 | **GHGIIIFLKNVNVFPIFL** | **8** | **25** | **18** | **HLA-DRB1*15:01** | **0.4476** | **A** |
| 63 | **YGHGIIIFLKNVNV** | **7** | **21** | **15** | **HLA-DRB1*15:01** | **0.4445** | **A** |
| 64 | **GHGIIIFLKNVNVFPIF** | **8** | **24** | **17** | **HLA-DRB1*15:01** | **0.4438** | **A** |
| 65 | **YGHGIIIFLKNVNVFPIFL** | **7** | **25** | **19** | **HLA-DRB1*15:01** | **0.4376** | **A** |
| 66 | **YGHGIIIFLKNVNVFPIF** | **7** | **24** | **18** | **HLA-DRB1*15:01** | **0.4327** | **A** |
| 67 | **FYHAGSSRLLTVGNPYFR** | **60** | **77** | **18** | **HLA-DRB1*09:01** | **0.4208** | **A** |
| 68 | **FYHAGSSRLLTVGNPYFRV** | **60** | **78** | **19** | **HLA-DRB1*09:01** | **0.4** | **A** |
| 69 | GIIIFLKNVNVF | 10 | 21 | 12 | HLA-DRB1*15:01 | 0.397 | NA |
| 70 | GHGIIIFLKNVNVFP | 8 | 22 | 15 | HLA-DRB1*15:01 | 0.3819 | NA |
| 71 | YGHGIIIFLKNVNVFP | 7 | 22 | 16 | HLA-DRB1*15:01 | 0.3738 | NA |
| 72 | NIIYGHGIIIFLKNVNV | 4 | 20 | 17 | HLA-DRB1*15:01 | 0.3725 | NA |
| 73 | IIYGHGIIIFLKNVNVFPI | 5 | 23 | 19 | HLA-DRB1*15:01 | 0.3705 | NA |
| 74 | FYHAGSSRLLTVG | 60 | 72 | 13 | HLA-DRB1*09:01 | 0.3667 | NA |
| 75 | FYHAGSSRLLTVGN | 60 | 73 | 14 | HLA-DRB1*09:01 | 0.3486 | NA |
| 76 | FYHAGSSRLLTVGNPYF | 60 | 76 | 17 | HLA-DRB1*09:01 | 0.3305 | NA |
| 77 | SRTSIFYHAGSSRLLTVG | 55 | 72 | 18 | HLA-DRB1*09:01 | 0.2913 | NA |
| 78 | MSYIHSMNSSIL | 418 | 429 | 12 | HLA-DRB1*09:01 | 0.2875 | NA |
|  |  |  |  |  | HLA-DRB1*04:05 |  |  |
| 79 | IIYGHGIIIFLKNVNV | 5 | 20 | 16 | HLA-DRB1*15:01 | 0.2838 | NA |
| 80 | VSRTSIFYHAGSSRLLTVG | 54 | 72 | 19 | HLA-DRB1*09:01 | 0.2836 | NA |
| 81 | SRTSIFYHAGSSRLLTVGN | 55 | 73 | 19 | HLA-DRB1*09:01 | 0.2806 | NA |
| 82 | IFYHAGSSRLLTVGNPYFR | 59 | 77 | 19 | HLA-DRB1*09:01 | 0.2663 | NA |
| 83 | HGIIIFLKNVNVF | 9 | 21 | 13 | HLA-DRB1*15:01 | 0.2526 | NA |
| 84 | IYGHGIIIFLKNVNVFPI | 6 | 23 | 18 | HLA-DRB1*15:01 | 0.2513 | NA |
| 85 | MSYIHSMNSSILENWN | 418 | 433 | 16 | HLA-DRB1*04:05 | 0.2478 | NA |
|  |  |  |  |  | HLA-DRB1*09:01 |  |  |
| 86 | RTSIFYHAGSSRLLTVG | 56 | 72 | 17 | HLA-DRB1*09:01 | 0.2468 | NA |
| 87 | IIYGHGIIIFLKNVNVFP | 5 | 22 | 18 | HLA-DRB1*15:01 | 0.24 | NA |
| 88 | RTSIFYHAGSSRLLTVGN | 56 | 73 | 18 | HLA-DRB1*09:01 | 0.2386 | NA |
| 89 | FYHAGSSRLLTVGNP | 60 | 74 | 15 | HLA-DRB1*09:01 | 0.2374 | NA |
| 90 | MSYIHSMNSSILE | 418 | 430 | 13 | HLA-DRB1*04:05 | 0.2305 | NA |
|  |  |  |  |  | HLA-DRB1*09:01 |  |  |
|  |  |  |  |  | HLA-DRB1*13:02 |  |  |
| 91 | FYHAGSSRLLTVGNPY | 60 | 75 | 16 | HLA-DRB1*09:01 | 0.2135 | NA |
| 92 | LTAEVMSYIHSMNSSIL | 413 | 429 | 17 | HLA-DRB1*09:01 | 0.2111 | NA |
| 93 | IYGHGIIIFLKNVNVFPIF | 6 | 24 | 19 | HLA-DRB1*15:01 | 0.1922 | NA |
| 94 | TSIFYHAGSSRLLTVG | 57 | 72 | 16 | HLA-DRB1*09:01 | 0.189 | NA |
| 95 | TSIFYHAGSSRLLTVGN | 57 | 73 | 17 | HLA-DRB1*09:01 | 0.1847 | NA |
| 96 | IFYHAGSSRLLTVGNPYF | 59 | 76 | 18 | HLA-DRB1*09:01 | 0.1732 | NA |
| 97 | TAEVMSYIHSMNSSIL | 414 | 429 | 16 | HLA-DRB1*09:01 | 0.1701 | NA |
|  |  |  |  |  | HLA-DRB1*04:05 |  |  |
| 98 | SIFYHAGSSRLLTVGNPYF | 58 | 76 | 19 | HLA-DRB1*09:01 | 0.1695 | NA |
| 99 | RTSIFYHAGSSRLLTVGNP | 56 | 74 | 19 | HLA-DRB1*09:01 | 0.165 | NA |
| 100 | MSYIHSMNSSILEN | 418 | 431 | 14 | HLA-DRB1*04:05 | 0.1541 | NA |
|  |  |  |  |  | HLA-DRB1*09:01 |  |  |
|  |  |  |  |  | HLA-DRB1*13:02 |  |  |
| 101 | IFYHAGSSRLLTVGN | 59 | 73 | 15 | HLA-DRB1*09:01 | 0.1499 | NA |
| 102 | IFYHAGSSRLLTVG | 59 | 72 | 14 | HLA-DRB1*09:01 | 0.1498 | NA |
| 103 | SIFYHAGSSRLLTVG | 58 | 72 | 15 | HLA-DRB1*09:01 | 0.1451 | NA |
| 104 | SIFYHAGSSRLLTVGN | 58 | 73 | 16 | HLA-DRB1*09:01 | 0.145 | NA |
| 105 | TAEVMSYIHSMNSSILE | 414 | 430 | 17 | HLA-DRB1*09:01 | 0.1381 | NA |
|  |  |  |  |  | HLA-DRB1*04:05 |  |  |
| 106 | IYGHGIIIFLKNVNV | 6 | 20 | 15 | HLA-DRB1*15:01 | 0.1271 | NA |
| 107 | YGHGIIIFLKNVNVF | 7 | 20 | 14 | HLA-DRB1*15:01 | 0.1228 | NA |
| 108 | AEVMSYIHSMNSSI | 415 | 428 | 14 | HLA-DRB1*04:05 | 0.1227 | NA |
| 109 | SYIHSMNSSILENWN | 419 | 433 | 15 | HLA-DRB1*09:01 | 0.1167 | NA |
| 110 | NIIYGHGIIIFLKNVNVF | 4 | 21 | 18 | HLA-DRB1*15:01 | 0.1145 | NA |
| 111 | VMSYIHSMNSSILENWN | 417 | 433 | 17 | HLA-DRB1*04:05 | 0.1138 | NA |
| 112 | GHGIIIFLKNVNVF | 8 | 21 | 14 | HLA-DRB1*15:01 | 0.1091 | NA |
| 113 | TSIFYHAGSSRLLTVGNP | 57 | 74 | 18 | HLA-DRB1*09:01 | 0.109 | NA |
| 114 | AEVMSYIHSMNSSIL | 415 | 429 | 15 | HLA-DRB1*09:01 | 0.1078 | NA |
|  |  |  |  |  | HLA-DRB1*04:05 |  |  |
|  |  |  |  |  | HLA-DRB1*13:02 |  |  |
| 115 | IYGHGIIIFLKNVNVFP | 6 | 22 | 17 | HLA-DRB1*15:01 | 0.1038 | NA |
| 116 | VMSYIHSMNSSIL | 417 | 429 | 13 | HLA-DRB1*09:01 | 0.0975 | NA |
|  |  |  |  |  | HLA-DRB1*04:05 |  |  |
|  |  |  |  |  | HLA-DRB1*13:02 |  |  |
| 117 | TSIFYHAGSSRLLTVGNPY | 57 | 75 | 19 | HLA-DRB1*09:01 | 0.0974 | NA |
| 118 | EVMSYIHSMNSSI | 416 | 428 | 13 | HLA-DRB1*04:05 | 0.0889 | NA |
| 119 | AEVMSYIHSMNSSILE | 415 | 430 | 16 | HLA-DRB1*09:01 | 0.0779 | NA |
|  |  |  |  |  | HLA-DRB1*04:05 |  |  |
| 120 | EVMSYIHSMNSSIL | 416 | 429 | 14 | HLA-DRB1*09:01 | 0.0777 | NA |
|  |  |  |  |  | HLA-DRB1*04:05 |  |  |
|  |  |  |  |  | HLA-DRB1*13:02 |  |  |
| 121 | SIFYHAGSSRLLTVGNP | 58 | 74 | 17 | HLA-DRB1*09:01 | 0.0662 | NA |
| 122 | IFYHAGSSRLLTVGNP | 59 | 74 | 16 | HLA-DRB1*09:01 | 0.0633 | NA |
| 123 | VMSYIHSMNSSILE | 417 | 430 | 14 | HLA-DRB1*09:01 | 0.0627 | NA |
|  |  |  |  |  | HLA-DRB1*04:05 |  |  |
|  |  |  |  |  | HLA-DRB1*13:02 |  |  |
| 124 | HNIIYGHGIIIFLKNVNVF | 3 | 21 | 19 | HLA-DRB1*15:01 | 0.058 | NA |
| 125 | SYIHSMNSSILE | 419 | 430 | 12 | HLA-DRB1*09:01 | 0.057 | NA |
| 126 | SIFYHAGSSRLLTVGNPY | 58 | 75 | 18 | HLA-DRB1*09:01 | 0.0569 | NA |
| 127 | MSYIHSMNSSILENW | 418 | 432 | 15 | HLA-DRB1*04:05 | 0.0568 | NA |
|  |  |  |  |  | HLA-DRB1*09:01 |  |  |
|  |  |  |  |  | HLA-DRB1*13:02 |  |  |
| 128 | IFYHAGSSRLLTVGNPY | 59 | 75 | 17 | HLA-DRB1*09:01 | 0.0533 | NA |
| 129 | EVMSYIHSMNSSILE | 416 | 430 | 15 | HLA-DRB1*04:05 | 0.0476 | NA |
|  |  |  |  |  | HLA-DRB1*09:01 |  |  |
|  |  |  |  |  | HLA-DRB1*13:02 |  |  |
| 130 | YVSRTSIFYHAGSSRLLTV | 53 | 71 | 19 | HLA-DRB1*09:01 | 0.0455 | NA |
| 131 | AEVMSYIHSMNSSILEN | 415 | 431 | 17 | HLA-DRB1*09:01 | 0.0262 | NA |
|  |  |  |  |  | HLA-DRB1*04:05 |  |  |
| 132 | IIYGHGIIIFLKNVNVF | 5 | 21 | 17 | HLA-DRB1*15:01 | 0.0153 | NA |
| 133 | VMSYIHSMNSSILEN | 417 | 431 | 15 | HLA-DRB1*04:05 | 0.0052 | NA |
|  |  |  |  |  | HLA-DRB1*09:01 |  |  |
|  |  |  |  |  | HLA-DRB1*13:02 |  |  |
| 134 | DYVSRTSIFYHAGSSRLL | 52 | 69 | 18 | HLA-DRB1*09:01 | -0.0038 | NA |
| 135 | EVMSYIHSMNSSILEN | 416 | 431 | 16 | HLA-DRB1*04:05 | -0.0052 | NA |
|  |  |  |  |  | HLA-DRB1*09:01 |  |  |
|  |  |  |  |  | HLA-DRB1*13:02 |  |  |
|  |  |  |  |  | HLA-DRB1*04:03 |  |  |
|  |  |  |  |  | HLA-DRB1*04:06 |  |  |
| 136 | VSRTSIFYHAGSSRLLTV | 54 | 71 | 18 | HLA-DRB1*09:01 | -0.0057 | NA |
| 137 | SYIHSMNSSILEN | 419 | 431 | 13 | HLA-DRB1*09:01 | -0.0092 | NA |
|  |  |  |  |  | HLA-DRB1*04:05 |  |  |
| 138 | SRTSIFYHAGSSRLLTV | 55 | 71 | 17 | HLA-DRB1*09:01 | -0.018 | NA |
| 139 | YVSRTSIFYHAGSSRLL | 53 | 69 | 17 | HLA-DRB1*09:01 | -0.0388 | NA |
| 140 | DYVSRTSIFYHAGSSRLLT | 52 | 70 | 19 | HLA-DRB1*09:01 | -0.058 | NA |
| 141 | DDYVSRTSIFYHAGSSRLL | 51 | 69 | 19 | HLA-DRB1*09:01 | -0.0665 | NA |
| 142 | VMSYIHSMNSSILENW | 417 | 432 | 16 | HLA-DRB1*04:05 | -0.0728 | NA |
|  |  |  |  |  | HLA-DRB1*09:01 |  |  |
|  |  |  |  |  | HLA-DRB1*13:02 |  |  |
| 143 | EVMSYIHSMNSSILENW | 416 | 432 | 17 | HLA-DRB1*04:05 | -0.0764 | NA |
| 144 | RTSIFYHAGSSRLLTV | 56 | 71 | 16 | HLA-DRB1*09:01 | -0.0896 | NA |
| 145 | YVSRTSIFYHAGSSRLLT | 53 | 70 | 18 | HLA-DRB1*09:01 | -0.0951 | NA |
| 146 | SYIHSMNSSILENW | 419 | 432 | 14 | HLA-DRB1*09:01 | -0.1023 | NA |
|  |  |  |  |  | HLA-DRB1*04:05 |  |  |
| 147 | VSRTSIFYHAGSSRLL | 54 | 69 | 16 | HLA-DRB1*09:01 | -0.1046 | NA |
| 148 | FYHAGSSRLLTV | 60 | 71 | 12 | HLA-DRB1*09:01 | -0.1086 | NA |
| 149 | SRTSIFYHAGSSRLL | 55 | 69 | 15 | HLA-DRB1*09:01 | -0.1267 | NA |
| 150 | IYGHGIIIFLKNVNVF | 6 | 21 | 16 | HLA-DRB1*15:01 | -0.1483 | NA |
| 151 | VSRTSIFYHAGSSRLLT | 54 | 70 | 17 | HLA-DRB1*09:01 | -0.16 | NA |
| 152 | TSIFYHAGSSRLLTV | 57 | 71 | 15 | HLA-DRB1*09:01 | -0.18 | NA |
| 153 | SRTSIFYHAGSSRLLT | 55 | 70 | 16 | HLA-DRB1*09:01 | -0.1848 | NA |
| 154 | RTSIFYHAGSSRLL | 56 | 69 | 14 | HLA-DRB1*09:01 | -0.2201 | NA |
| 155 | SIFYHAGSSRLLTV | 58 | 71 | 14 | HLA-DRB1*09:01 | -0.2624 | NA |
| 156 | RTSIFYHAGSSRLLT | 56 | 70 | 15 | HLA-DRB1*09:01 | -0.2758 | NA |
| 157 | IFYHAGSSRLLTV | 59 | 71 | 13 | HLA-DRB1*09:01 | -0.298 | NA |
| 158 | TSIFYHAGSSRLL | 57 | 69 | 13 | HLA-DRB1*09:01 | -0.3401 | NA |
| 159 | TSIFYHAGSSRLLT | 57 | 70 | 14 | HLA-DRB1*09:01 | -0.3906 | NA |
| 160 | SIFYHAGSSRLL | 58 | 69 | 12 | HLA-DRB1*09:01 | -0.4605 | NA |
| 161 | SIFYHAGSSRLLT | 58 | 70 | 13 | HLA-DRB1*09:01 | -0.503 | NA |
| 162 | IFYHAGSSRLLT | 59 | 70 | 12 | HLA-DRB1*09:01 | -0.5684 | NA |

Supplementary Table 6 The population coverage data of the selected HLA-1 and HLA-2 epitope in combined form.

|  | Country | Coverage |  | Country | Coverage |
| --- | --- | --- | --- | --- | --- |
| 1 | Algeria | 9.62% | 49 | Macedonia | 7.49% |
| 2 | American Samoa | 55.59% | 50 | Malaysia | 25.55% |
| 3 | Argentina | 27.53% | 51 | Mali | 26.29% |
| 4 | Australia | 44.27% | 52 | Martinique | 7.62% |
| 5 | Austria | 45.07% | 53 | Mongolia | 19.07% |
| 6 | Belarus | 19.77% | 54 | Morocco | 25.61% |
| 7 | Belgium | 51.22% | 55 | Nauru | 7.26% |
| 8 | Borneo | 0.00% | 56 | Netherlands | 13.10% |
| 9 | Brazil | 24.84% | 57 | New Caledonia | 34.37% |
| 10 | Bulgaria | 27.96% | 58 | New Zealand | 9.38% |
| 11 | Burkina Faso | 10.43% | 59 | Niue | 0.00% |
| 12 | Cameroon | 24.57% | 60 | Norway | 26.04% |
| 13 | Canada | 1.12% | 61 | Oman | 15.25% |
| 14 | Cape Verde | 32.22% | 62 | Pakistan | 28.32% |
| 15 | Central African Republic | 46.99% | 63 | Papua New Guinea | 40.73% |
| 16 | Chile | 34.35% | 64 | Paraguay | 0.00% |
| 17 | China | 29.99% | 65 | Peru | 3.39% |
| 18 | Colombia | 2.62% | 66 | Philippines | 52.39% |
| 19 | Congo | 2.19% | 67 | Poland | 41.62% |
| 20 | Cook Islands | 6.06% | 68 | Portugal | 29.62% |
| 21 | Croatia | 33.74% | 69 | Romania | 31.95% |
| 22 | Cuba | 32.96% | 70 | Russia | 55.65% |
| 23 | Czech Republic | 46.44% | 71 | Rwanda | 3.55% |
| 24 | Denmark | 33.58% | 72 | Samoa | 1.99% |
| 25 | England | 52.95% | 73 | Sao Tome and Principe | 22.47% |
| 26 | Ethiopia | 3.25% | 74 | Saudi Arabia | 30.83% |
| 27 | Fiji | 0.00% | 75 | Scotland | 24.76% |
| 28 | Finland | 62.56% | 76 | Senegal | 24.06% |
| 29 | France | 49.42% | 77 | Serbia | 9.75% |
| 30 | Georgia | 33.84% | 78 | Singapore | 37.39% |
| 31 | Germany | 50.56% | 79 | Slovenia | 11.49% |
| 32 | Greece | 6.66% | 80 | South Africa | 15.65% |
| 33 | Guatemala | 0.80% | 81 | Spain | 22.09% |
| 34 | Guinea-Bissau | 26.80% | 82 | Sudan | 20.44% |
| 35 | Hong Kong | 44.05% | 83 | Sweden | 72.16% |
| 36 | India | 34.00% | 84 | Taiwan | 52.44% |
| 37 | Indonesia | 40.18% | 85 | Thailand | 17.48% |
| 38 | Iran | 27.50% | 86 | Tunisia | 28.92% |
| 39 | Ireland Northern | 50.56% | 87 | Turkey | 8.99% |
| 40 | Ireland South | 43.44% | 88 | Uganda | 19.05% |
| 41 | Israel | 33.60% | 89 | Ukraine | 21.22% |
| 42 | Italy | 31.65% | 90 | United Arab Emirates | 8.99% |
| 43 | Jamaica | 6.68% | 91 | United States | 43.50% |
| 44 | Jordan | 33.09% | 92 | Venezuela | 37.35% |
| 45 | Kenya | 10.28% | 93 | Vietnam | 33.59% |
| 46 | Kiribati | 0.00% | 94 | Wales | 2.78% |
| 47 | Korea; South | 49.63% | 95 | Zambia | 15.27% |
| 48 | Lebanon | 19.36% | 96 | Zimbabwe | 13.09% |
| Average | | | | | 25.65% |
| Standard deviation | | | | | 16.99% |
